# Uniform two-dimensional square assemblies from conjugated block copolymers driven by π–π interactions with controllable sizes

**DOI:** 10.1038/s41467-018-03195-y

**Published:** 2018-02-28

**Authors:** Liang Han, Meijing Wang, Xiangmeng Jia, Wei Chen, Hujun Qian, Feng He

**Affiliations:** 1 Department of Chemistry, Southern University of Science and Technology, Shenzhen, 518055 China; 2 State Key Laboratory of Supramolecular Structure and Materials, Institute of Theoretical Chemistry, Jilin University, Changchun, 130023 China; 3 Materials Science Division, Argonne National Laboratory, 9700 Cass Avenue, Lemont, IL 60439 USA; 4 Institute of Molecular Engineering, The University of Chicago, 5640 South Ellis Avenue, Chicago, IL 60637 USA

## Abstract

Two-dimensional (2-D) micro- and nano-architectures are attractive because of their unique properties. However, the formation of 2-D supramolecular highly symmetrical structures with considerable control is still a major challenge. Here we present a simple approach for the preparation of regular and homogeneous 2-D fluorescent square non-crystallization micelles with conjugated diblock copolymers PPV_12_-*b*-P2VP_*n*_ through a process of dissolving–cooling–aging. The scale of the formed micelles can be controlled by the ratio of PPV/P2VP blocks and the concentration of the solution. The results reveal that the micelles of PPV_12_-*b*-P2VP_*n*_ initially form 1-D structures and then grow into 2-D structures in solution, and the growth is driven by intermolecular π–π interactions with the PPV_12_ blocks. The formation of 2-D square micelles is induced by herringbone arrangement of the molecules, which is closely related to the presence of the branched alkyl chains attached to conjugated PPV_12_ cores.

## Abstract (web summary)

Crystallization-driven processes play a vital role in preparing 2D nanostructures which makes structures with high symmetry hard to access. Here the authors present a non-crystallization approach which is based on π–π interactions of a copolymer for the fabrication of 2D symmetric structures with good dimensional control.

## Introduction

Recently, nanoscale two-dimensional (2-D) self-assembled architectures have attracted a great deal of attention as a result of their unique properties caused by their ultrathin and flat morphologies^1,2^. Over the past few years, various 2-D micro- and nano-structures have been constructed from inorganic metal salts^3^, organic molecules^4^, proteins^5,6^, and block copolymers (BCPs)^7–9^. Crystallization-driven processes have a vital role in the methods of preparing 2-D assembled nanostructures and the shapes of these formed 2-D nanostructures are thus controlled by the symmetry of the unit cell. Therefore, 2-D micro- and nano-architectures with high symmetry, including equilateral triangular, square-shaped, hexagonal, and circular assemblies, are still not commonly reported. As the creation of synthetic 2-D materials represents an attractive challenge that is ultimately driven by their prospective uses in, e.g., electronics, biomedicine, catalysis, sensing, and as membranes for separation and filtration^1^, the acquisition of uniform 2-D assemblies with regular shapes has more important significance. Achieving such a uniform architecture through non-crystallization-derived approaches^10^ such as Van der Waals’ forces, hydrophilic/hydrophobic interactions, hydrogen bonds, and π–π interactions is particularly challenging and fascinating.

A bottom-up self-assembly of crystalline homopolymers and BCPs is a commonly used strategy to prepare 2-D materials. In recent years, a series of analogous polymers have been studied for 2-D self-assembly and colloidally stable 2-D nanostructures were formed in solution^11–13^. The classic poly(ferrocenyldimethylsilane) BCPs have been reported to yield unique 2-D lenticular and rectangular platelet block co-micelles with semi-crystalline properties by a seeded growth processes^14–16^. However, it is difficult to substantially control the structures of the 2-D BCP micelles and non-crystalline regular 2-D architectures are still rarely obtained. Generating uniform colloidally stable 2-D micro- and nano- structures with controlling their shapes and dimensions remains a key challenge.

Poly(p-phenylenevinylene) (PPV) is a classic type of π-conjugated material with fascinating optical and electronic properties^17,18^. The long conjugated backbones allow PPV to achieve tight intermolecular packing^19^ with intense sheet-forming assembling tendency. However, although a certain number of PPV-based BCPs have been utilized as building blocks for supramolecular self-assembly^20–23^, few of them, which were always irregular one-dimensional (1-D) cylindrical or fibrous, took advantage of the packing inclination to achieve uniform 2-D aggregates^24^.

In this study, we combine PPV blocks and poly (2-vinyl pyridine) (P2VP) to build amphiphilic diblock copolymers PPV_12_-*b*-P2VP_*n*_ with sheet–coil-type structures, and colloidally stable 2-D fluorescent square micelles are formed in certain solvents, such as isopropanol, with easy approaches. Low dispersities and good dimensional control over the 2-D square micelles are achieved by adjusting the ratio of P2VP and PPV blocks, as well as the concentration of the solution. Furthermore, it is found that the assembly process of 2-D micelles in solution is closely related to the temperature. Intermolecular π–π interactions and the herringbone molecular arrangement have vital roles in the formation of square micelle films. In view of the perfect optical and electronic properties of PPV blocks, the forming square micelles should have potential applications in the fields of organic field effect transistors (OFET), organic photovoltaic (OPV), and biosensing.

## Results

### Design and preparation of π-conjugated BCP micelles

To use π–π interactions to form self-assembly architectures, we built a series of supramolecular BCPs composed of a core-forming π-conjugated block of poly(2,5-di(2′-ethylhexyloxy)-1,4-phenylenevinylene) (PPV) and a corona block of P2VP. The PPV-aldehyde homopolymer was prepared by Siegrist polycondensation and the monomer number of PPV block was calculated to be 12 by nuclear magnetic resonance (NMR), whose polydispersity was 1.05 determined by gel permeation chromatography (GPC). Then a series of diblock copolymers PPV_12_-*b*-P2VP_*n*_ (the subscript *n* describes the number-average degrees of polymerization) were synthesized through quenching the anionic polymerization of P2VP with the PPV aldehyde. The degrees of polymerization *n* was 12, 16, 22, 36, 46, and 71 (*R*_PPV/P2VP_, which was short for the block ratio of PPV and P2VP, was nearly 1 : 1, 1 : 1.3, 1 : 2, 1 : 3, 1 : 4, and 1 : 6), respectively, characterized by NMR and GPC (see Supplementary Methods, Supplementary Figs. 1–12, and Table 1).

The obtained BCPs PPV_12_-*b*-P2VP_*n*_ are typically amphiphilic, so they should readily assemble into ordered micelles in solutions. To confirm whether the π–π interactions of PPV_12_ blocks could take effect in the formation of the simple micelles, we attempted to dissolve the BCPs PPV_12_-*b*-P2VP_*n*_ in 2-propanol (2-PrOH), which is a selective solvent for P2VP coronas. The solution was heated at 85 °C for 45 min, then it was slowly cooled to room temperature (25 °C) followed by aging for 24 h. Interestingly, uniform regular 2-D square micelles of PPV_12_-*b*-P2VP_*n*_ were yielded from the resulting colloidally stable solutions by this simple approach, which was observed under transmission electron microscopy (TEM).

### Tuning the scale of the square micelles by varying building block ratios and solution concentrations

**Fig. 1:**
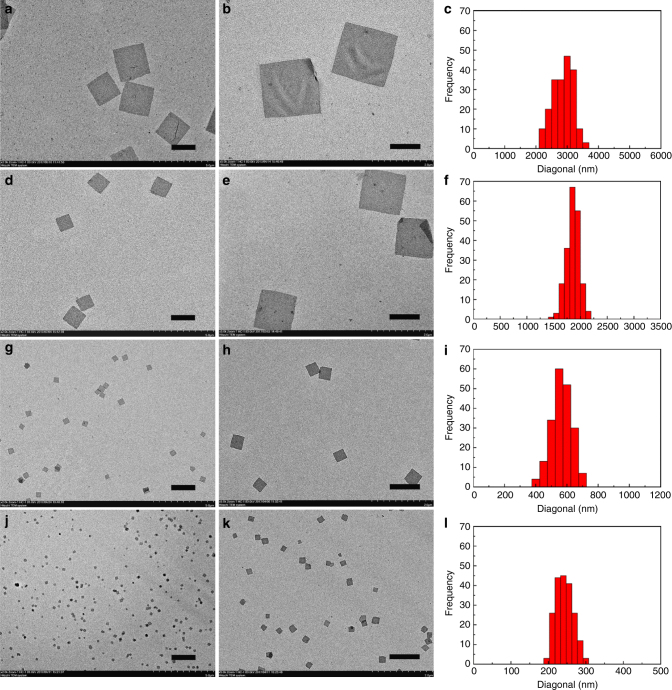
2-D square micelles grown from PPV_12_-*b*-P2VP_*n*_ with varying ratio of PPV and P2VP blocks (*R*_PPV/P2VP_). TEM images of representative sample of 2-D square micelles prepared from **a**,** b** 0.01 mg ml^−1^ 2-PrOH solution of PPV_12_-*b*-P2VP_12_ (*R*_PPV/P2VP_ = 1 : 1), **d**,** e** 0.01 mg ml^−1^ 2-PrOH solution of PPV_12_-*b*-P2VP_16_ (*R*_PPV/P2VP_ = 1 : 1.3), **g**,** h** 0.05 mg ml^−1^ 2-PrOH solution of PPV_12_-*b*-P2VP_22_ (*R*_PPV/P2VP_ = 1 : 2), **j**,** k** 0.05 mg ml^−1^ 2-PrOH solution of PPV_12_-*b*-P2VP_36_ (*R*_PPV/P2VP_ = 1 : 3), and **c**, **f**, **i**, **l** contour length distributions of the diagonal length of correspondent 2-D square micelles. Scale bars in images **a**, **d**, **g**, **j** are 2 μm, whereas scale bars in images **b**, **e**, **h**, **k** are 1 μm

It was observed that the scales and morphologies of the achieved 2-D micelles were influenced by the ratio of PPV/P2VP blocks (*R*_PPV/P2VP_). The number-average diagonal length (*D*_n_, the subscripts refer to the number-average diagonal length) of the square micelles formed in a 0.01 mg ml^−1^ solution of PPV_12_-*b*-P2VP_12_ (*R*_PPV/P2VP_ = 1 : 1) in 2-PrOH was calculated to be 2,747 nm (Fig. 1a,b and Supplementary Fig. 13a) and the diagonal length dispersity *D*_w_/*D*_n_ = 1.02 (*D*_w _= 2,788 nm, *D*_w_ is the weight-average diagonal length, Fig. 1c). These square micelles were highly flexible and they were easily cracked and wrinkled because of membrane mechanics (Supplementary Fig. 14a); these features were clearly observed by scanning electron microscope (SEM) (Supplementary Fig. 16). Meanwhile, the large square micelles readily packed with each other. As the value of *R*_PPV/P2VP_ was reduced, the scales of the formed square micelles clearly decreased, and the uniformity and integrity of the micelles improved. *D*_n_ of square micelles of PPV_12_-*b*-PPV_16_ (*R*_PPV/P2VP_ = 1 : 1.3, 0.01 mg ml^−1^ in 2-PrOH solution) reduced to 1,808 nm (*D*_w_ = 1,815 nm, *D*_w_/*D*_n_ = 1.00, Fig. 1d–f, and Supplementary Fig. 13b and 14b). When *R*_PPV/P2VP_ was reduced to nearly 1 : 2, the obtained square micelles of PPV_12_-*b*-P2VP_22_ from 0.05 mg ml^−1^ 2-PrOH solution showed decreased scales (*D*_n_ = 542 nm), improved homogeneity (*D*_w_/D_n_ = 1.02, *D*_*w*_ = 551 nm), and more uniform morphologies (Fig. 1g–i and Supplementary Fig. 14c). While the *D*_n_ of square micelles of PPV_12_-*b*-P2VP_36_ (*R*_PPV/P2VP_ = 1 : 3, prepared in 0.05 mg ml^−1^ 2-PrOH solution) was only 235 nm with a narrow diagonal length dispersity (*D*_w_/*D*_n_ = 1.01, *D*_w_ = 238 nm, Fig. 1j–l and Supplementary Fig. 14d). When *R*_PPV/P2VP_ was reduced to 1 : 4, 2-D supramolecular architectures were also achieved from 2-PrOH solution, but the scales of the formed nanostructures were not smaller than those of PPV_12_-*b*-P2VP_36_ and the morphologies were no longer regular squares (Supplementary Fig 15a, b). In addition, the PPV_12_-*b*-P2VP_71_ (*R*_PPV/P2VP_ = 1 : 6) assembled into noticeably smaller irregular dot-like 2-D nanostructures (Supplementary Fig 15c, d).

**Fig. 2:**
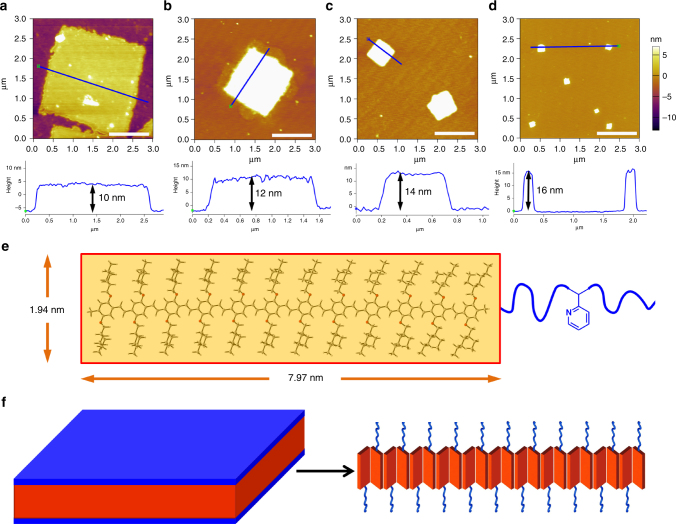
Probable conformation of platelet 2-D square micelles. AFM images of 2-D square micelles obtained from **a** 0.01 mg ml^−1^ 2-PrOH solution of PPV_12_-*b*-P2VP_12_, **b** 0.01 mg ml^−1^ 2-PrOH solution of PPV_12_-*b*-P2VP_16_, **c** 0.05 mg ml^−1^ 2-PrOH solution of PPV_12_-*b*-P2VP_22_, **d** 0.05 mg ml^−1^ 2-PrOH solution of PPV_12_-*b*-P2VP_36_ in the height mode. Scale bars correspond to 1 μm in AFM images. **e** The optimized structure and calculated scales of conjugated PPV_12_ block by DFT calculations. The DFT calculation was performed with the Gaussian09 suite of programs. The structure was optimized at the B3LYP level of theory with the 6-31G* basis set. **f** Schematic representation and probable molecular packing of the 2-D square micelles formed by assembly of PPV_12_-*b*-P2VP_*n*_

The heights of the 2-D square micelles of the copolymers with *R*_PPV/P2VP_ of 1 : 1, 1 : 1.3, 1 : 2, and 1 : 3 were calculated to be 10, 12, 14, and 16 nm (Fig. 2a–d), respectively, using atomic force microscopy (AFM). It was obvious that the thickness of the formed platelet 2-D square micelles of PPV_12_-*b*-P2VP_*n*_ increased as the length of the corona of P2VP was increased. Furthermore, the structure of the conjugated PPV_12_ block and P2VP_*n*_ blocks were optimized by density functional theory (DFT) calculations with the Gaussian09 suite^25^ of programs at the B3LYP level of theory^26,27^ with the 6-31G* basis set^28–30^. The PPV_12_ block exhibited a rigid skeleton and its length was calculated as 7.97 nm (Fig. 2e), whereas the length of the coronas of the P2VP_12_, P2VP_16_, P2VP_22_, and P2VP_36_ blocks were estimated to be 2.97, 3.86, 5.33, and 8.70 nm, respectively (Supplementary Fig. 17). The flexible P2VP coronas are probably folded and twined on the upper and lower surfaces of the formed platelet 2-D square micelles, and a single layer of PPV_12_ blocks packing with each other by π–π interactions are sandwiched in as illustrated in Fig. 2f, and these conformations are consistent with the individual thicknesses of the 2-D square micelles determined from AFM measurements.

**Fig. 3:**
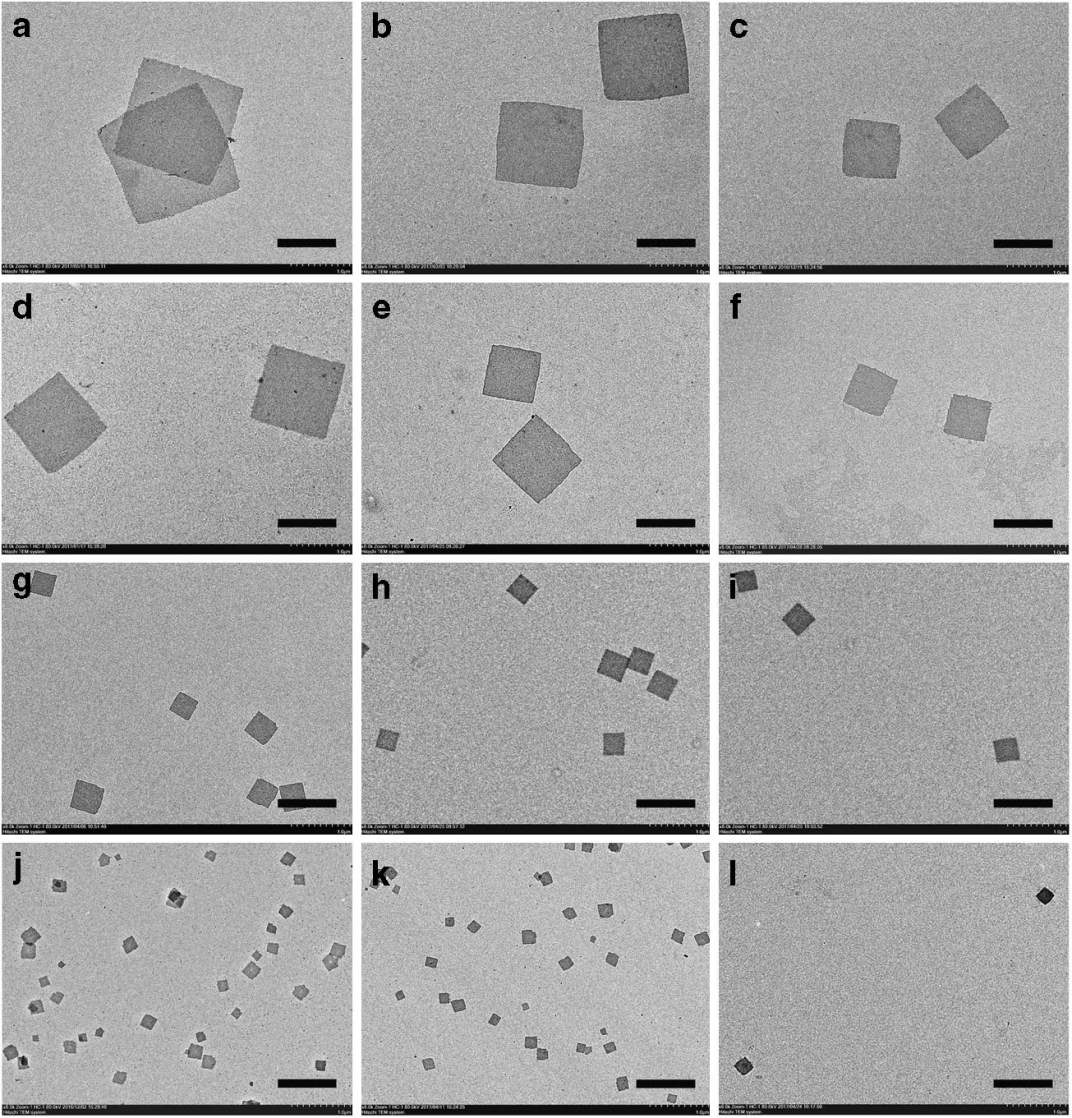
Concentration dependence of the formation of platelet 2-D square micelles. TEM images of representative samples of 2-D square micelles prepared from PPV_12_-*b*-P2VP_*n*_ with different *R*_PPV/P2VP_ in varying concentrations of solutions. **a**–**c** The representative 2-D square micelles of PPV_12_-*b*-P2VP_12_ (*R*_PPV/P2VP_ = 1 : 1) grew from the 2-PrOH solution of **a** 0.01 mg ml^−1^, **b** 0.005 mg ml^−1^, and **c** 0.0025 mg ml^−1^. **d**–**f** The representative 2-D square micelles of PPV_12_-*b*-P2VP_16_ (*R*_PPV/P2VP_ = 1 : 1.3) grew from the 2-PrOH solution of **d** 0.01 mg ml^−1^, **e** 0.005 mg ml^−1^, and **f** 0.0025 mg ml^−1^. **g**–**i** The representative 2-D square micelles of PPV_12_-*b*-P2VP_22_ (*R*_PPV/P2VP_ = 1  :2) grew from the 2-PrOH solution of **g** 0.05 mg ml^−1^, **h** 0.02 mg ml^−1^, and **i** 0.01 mg ml^−1^. **j**–**l** The representative 2D square micelles of PPV_12_-*b*-P2VP_36_ (*R*_PPV/P2VP_ = 1 : 3) grew from the 2-PrOH solution of **j** 0.05 mg ml^−1^, **k** 0.02 mg ml^−1^, and **l** 0.01 mg ml^−1^. Scale bars correspond to 1 μm in all TEM images

To control the scales of the achieved square micelles, we investigated the concentration dependence of the self-assembly of PPV_12_-*b*-P2VP_*n*_ and the results were quite interesting. The scales of the formed square micelles of PPV_12_-*b*-P2VP_12_ were significantly concentration dependent. The diagonal length of the square micelles formed in 0.01 mg ml^−1^ 2-PrOH solution was ~2,750 nm, whereas the diagonal length was reduced to ~2,000 nm when the concentration of the solution was decreased to 0.005 mg ml^−1^. When the solution concentration was halved again, the diagonal length of the formed square micelles further decreased to only ~1,300 nm (Fig. 3a–c). The obtained 2-D square micelles of PPV_12_-*b*-P2VP_16_ also showed concentration dependence, but the phenomenon was not as obvious as it was with the micelles of PPV_12_-*b*-P2VP_12_. The diagonal length of the square micelles formed in 2-PrOH solutions continued to decrease as the concentrations were halved from 0.01 all the way to 0.0025 mg ml^−1^; the diagonal lengths were ~ 1,950, 1,400, and 850 nm (Fig. 3d–f), respectively. When the P2VP blocks of the copolymer became longer, meaning that the *R*_PPV/P2VP_ value was <1 : 2, the concentration dependence of the self-assembly of PPV_12_-*b*-P2VP_*n*_ disappeared completely. For example, the diagonal lengths of the square micelles of PPV_12_-*b*-P2VP_22_ formed in 2-PrOH solution with concentrations of 0.05, 0.02, and 0.01 mg ml^−1^ were all ~550 nm with very little variation. Similarly, the diagonal lengths of the formed square micelles of PPV_12_-*b*-P2VP_36_ were only ~250 nm, which did not change when the concentration of the solution was decreased.

The formation of square micelles should be driven by both hydrophobic interaction and π-π interactions of the PPV_12_ blocks. The aggregation of the diblocks driven by hydrophobic interaction should be dynamic with regard to the exchange of the unimer building blocks, as reported previously^31^. As the dynamic process is significantly dependent on the concentration, the scales of 2-D square micelles formed by PPV_12_-*b*-P2VP_12_ and PPV_12_-*b*-P2VP_16_ with low *R*_PPV/P2VP_, whose dynamic exchanges were strong, were clearly influenced by the solution concentration. When the length of the P2VP corona was increased, the dynamic effect caused by hydrophobic interactions could be suppressed, so the process became slow enough compared with the time scale of the formation of the square platelet micelles. Therefore, the scales of the platelet square micelles of PPV_12_-*b*-P2VP_22_ and PPV_12_-*b*-P2VP_36_ with longer coronas did not vary as the solution concentration changed.

### Growth process of the platelet 2-D square micelles

**Fig. 4:**
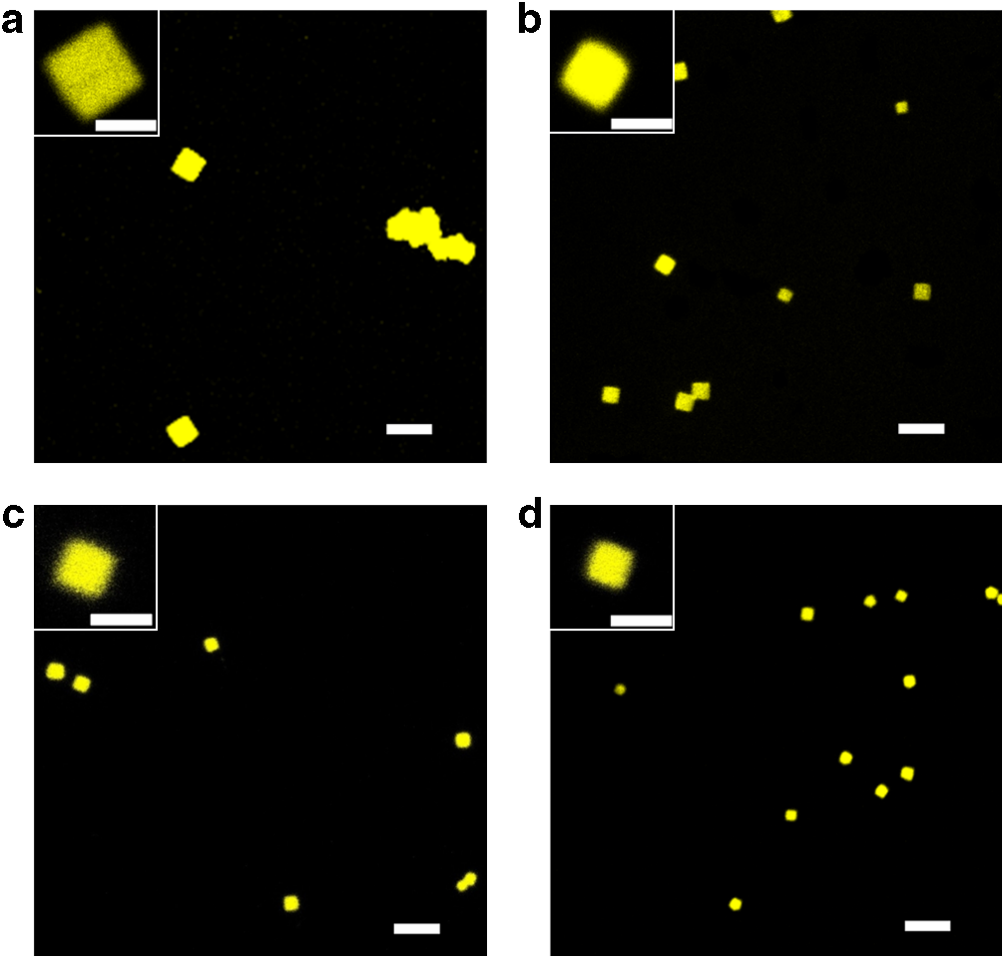
LSCM images of fluorescent platelet 2-D square micelles. LSCM images of representative samples of 2D square micelles in **a** 0.01 mg ml^−1^ 2-PrOH solution of PPV_12_-*b*-P2VP_12_; **b** 0.005 mg ml^−1^ 2-PrOH solution of PPV_12_-*b*-P2VP_12_; **c** 0.01 mg ml^−1^ 2-PrOH solution of PPV_12_-*b*-P2VP_16_, and **d** 0.005 mg ml^−1^ 2-PrOH solution of PPV_12_- *b*-P2VP_16_. Scale bars of all photos are 5 μm, whereas those of insets are 2 μm

Owing to the π-conjugated PPV_12_ blocks, the PPV_12_-*b*-P2VP_*n*_ copolymers displayed strong fluorescence in solutions. Compared with PPV_12_-*b*-P2VP_*n*_ in 0.01 mg ml^−1^ tetrahydrofuran (THF) solutions, where the copolymer molecules dissolved completely, PPV_12_-*b*-P2VP_*n*_, after aging in 0.01 mg ml^−1^ 2-PrOH solutions for 48 h, displayed blue-shifted emission peaks (at ~549 nm with a shoulder at 584 nm), broader photoluminescence (PL) and lower fluorescence quantum efficiency (*Φ*_F_, changed from 0.27 to 0.09) (Supplementary Fig 18). These changes originated from the formation of platelet 2-D square micelles in 2-PrOH solutions, which was induced by the aggregation of the copolymer molecules. The significant changes in fluorescence of PPV_12_-*b*-P2VP_12_ and PPV_12_-*b*-P2VP_16_ in solution are likely caused by the stacking of the large square micelles. In addition, if a drop of a solution of PPV_12_-*b*-P2VP_12_ or PPV_12_-*b*-P2VP_16_ that had been aged for 24 h was placed on a slide, the fluorescent square micelles could be clearly observed in solution by laser scanning confocal microscopy (LSCM). The *D*_n_ of the 2-D square micelles of PPV_12_-*b*-P2VP_12_ (*R*_PPV/P2VP_ = 1 : 1) in 0.01 and 0.005 mg ml^−1^ solution of 2-PrOH was calculated to be 3,200 nm and 2,300 nm, respectively, and those of PPV_12_-*b*-P2VP_16_ (*R*_PPV/P2VP_ = 1 : 1.3) in 0.01 and 0.005 mg ml^−1^ solution of 2-PrOH was calculated to be 2,200 and 1,700 nm. From the LSCM images, although the scales of the observed fluorescent square micelles were larger than those observed by TEM because of light scattering, the concentration dependence of the scales of 2-D square micelles formed by PPV_12_-*b*-P2VP_12_ and PPV_12_-*b*-P2VP_16_ with low *R*_PPV/P2VP_ were clearly observed, which was consistent with the results observed by TEM (Fig. 4). However, in a solution of PPV_12_-*b*-P2VP_22_ solution, only the fluorescent dot-like structures could be seen by LSCM, because the formed 2-D square micelles were too small to be clearly observed (Supplementary Fig 19). These observations from LSCM indicated that the self-assembly process of the formation of the 2-D square micelles occurred in the solution instead of occurring as the solvent evaporated, which had been further confirmed by the observation of the freely moving fluorescent aggregates in 0.01 mg ml^−1^ solutions of PPV_12_-*b*-P2VP_12_, although the square shape of the aggregates was unable to be clearly observed because of the scattering effect and the moving effect (Supplementary Movie 1).

**Fig. 5:**
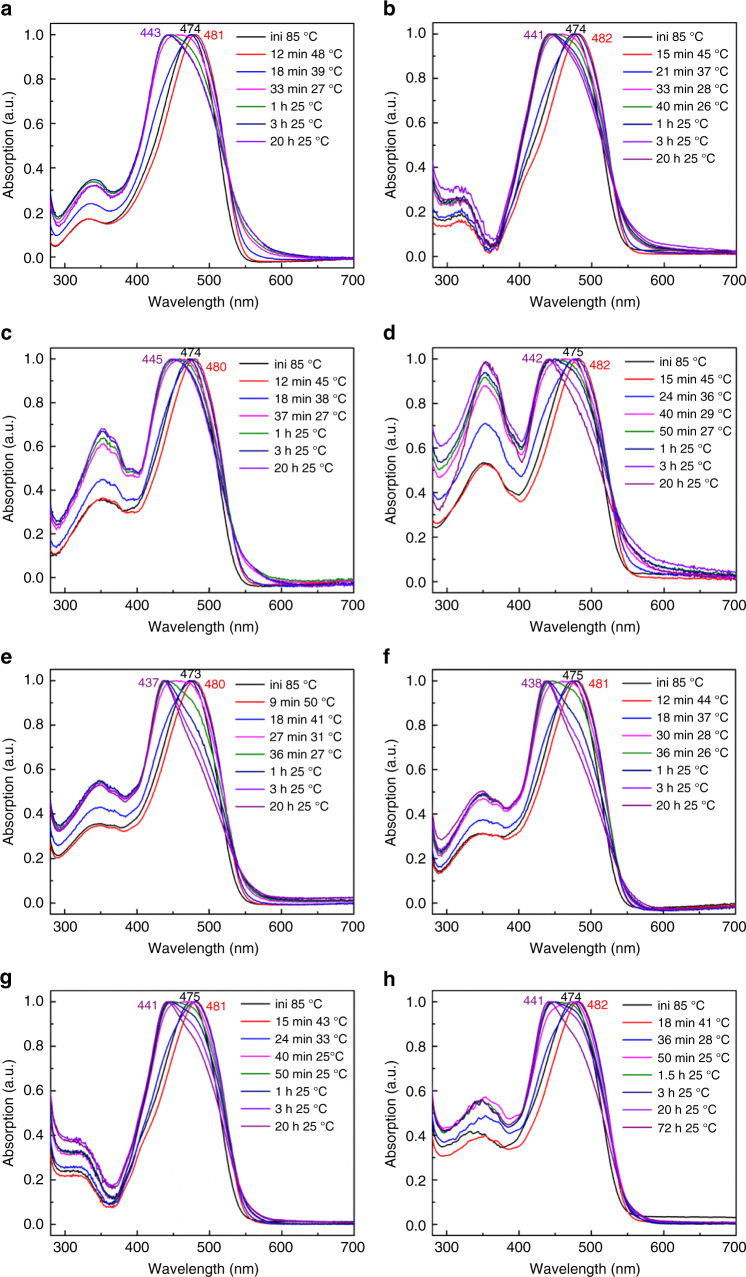
UV-Vis absorption changes of PPV_12_-*b*-P2VP_n_ in 2-PrOH solution. The varying UV-Vis absorption spectra of **a** PPV_12_-*b*-P2VP_12_ in 0.01 mg mL^−1^ 2-PrOH solution; **b** PPV_12_-*b*-P2VP_12_ in 0.0025 mg mL^−1^ 2-PrOH solution; **c** PPV_12_-*b*-P2VP_16_ in 0.01 mg ml^−1^ 2-PrOH solution; **d** PPV_12_-*b*-P2VP_16_ in 0.0025 mg ml^−1^ 2-PrOH solution; **e** PPV_12_-*b*-P2VP_22_ in 0.05 mg ml^−1^ 2-PrOH solution; **f** PPV_12_-*b*-P2VP_22_ in 0.01 mg ml^−1^ 2-PrOH solution; **g** PPV_12_-*b*-P2VP_36_ in 0.05 mg ml^−1^ 2-PrOH solution; and **h** PPV_12_-*b*-P2VP_36_ in 0.01 mg ml^−1^ 2-PrOH solution. All the sample solutions were heated at 85 °C for 30 min, then naturally cooled down to room temperature followed by aging for another ~ 20 h or more

To explore the assembly process of the BCPs from 2-PrOH solutions, we measured the UV-Vis absorption spectra of PPV_12_-*b*-P2VP_*n*_ in 2-PrOH as the temperature and the aging time changed (Fig. 5). All four PPV_12_-*b*-P2VP_*n*_ BCPs showed a peak at ~ 474 nm in the UV-Vis spectra in 2-PrOH solutions at 85 °C, whereas the molecules of PPV_12_-*b*-P2VP_*n*_ should completely dissolve in the solution and move freely. As the temperature of the solution decreased to 40~50 °C, the UV-Vis spectra of PPV_12_-*b*-P2VP_*n*_ exhibited slight bathochromic shifts of 6~8 nm, which indicated that the copolymer molecules had begun to aggregate in the solution because of the hydrophobic interactions of the conjugated core and the side alkyl chains. Then, the intensities of the spectra at 440~445 nm began to increase as the temperature was further decreased and the peaks almost disappeared when the solutions were cooled down to near room temperature. Finally, after aging for ~20 h, the peaks at ~440 nm appeared in the UV-Vis spectra of the copolymer solutions. The significant blue-shift of the absorption spectra suggested the formation of 2-D square micelles was driven by H-aggregation of the PPV_12_ blocks^21,23^. The UV-Vis spectra of PPV_12_-*b*-P2VP_*n*_ solutions all tended to change similarly as the solutions were cooled from 85 °C to room temperature, indicating that aggregation of the molecules was affected by the temperature. During the aging period at room temperature, the differences in the UV-Vis spectra of the four copolymers were substantially different from each other, suggesting their aggregating rates were different. For PPV_12_-*b*-P2VP_12_ and PPV_12_-*b*-P2VP_16_ with low *R*_PPV/P2VP_ values, the absorption intensities between 460 and 480 nm were lower in solutions that had aged for 1 h compared with those that had just been cooled to room temperature, and this deviation was caused by H-aggregation of the approaching molecules. After aging for 3 h, the intensities of the UV-Vis spectra between 460 and 480 nm continued to decline, but after aging for 20 h, the spectra of the high concentration solutions of PPV_12_-*b*-P2VP_12_ and PPV_12_-*b*-P2VP_16_ stopped changing; the intensities of the UV-Vis spectra of solutions with low concentrations decreased only a little in the 460~480 nm region compared with those of the solutions aged for 3 h. These changes may have occurred because square micelles of PPV_12_-*b*-P2VP_12_ and PPV_12_-*b*-P2VP_16_ with low *R*_PPV/P2VP_ formed from solutions with high concentration tended to pile with each other, and the piling effect became weaker in solutions of low concentration, which was consistent with the TEM observations. PPV_12_-*b*-P2VP_22_ in a 2-PrOH solution can assemble into homogeneous and regular 2-D square micelles with no concentration dependence. Therefore, the intensities of its UV-Vis spectra in the 460~480 nm region could decline completely after aging regardless of concentration. The trends observed in the intensities of the peaks in the UV-Vis spectra of PPV_12_-*b*-P2VP_36_ in solution were similar with those of low concentration solution spectra of PPV_12_-*b*-P2VP_16_. The incompletely declined intensities in the 460~480 nm region compared with PPV_12_-*b*-P2VP_22_ should be attributed to the partial H-aggregation caused by the longer P2VP coronas. The change in the spectra indicated that the aggregation rate of the PPV_12_-*b*-P2VP_36_ molecules was far slower than that of the other three copolymers; the complete aggregation of PPV_12_-*b*-P2VP_36_ molecules in 0.01 mg ml^−1^ 2-PrOH solution required 72 h of aging.

**Fig. 6:**
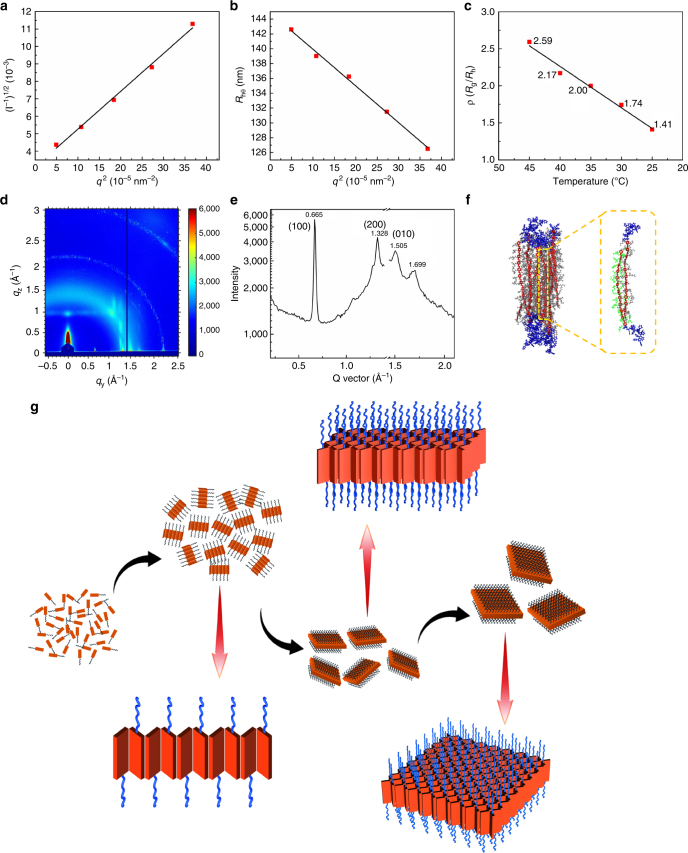
Assembling process of PPV_12_-*b*-P2VP_*n*_ in 2-PrOH solution. **a**,** b** The morphology factor *R*_g_/*R*_h_ (*ρ*) of PPV_12_-*b*-P2VP_22_ in 0.05 mg ml^−1^ 2-PrOH solution after aging for 2 days was calculated to be 1.36 by DLS and SLS. The root mean square radius of gyration (*R*_g_) is calculated by measuring SLS results of PPV_12_-*b*-P2VP_22_ in 0.05 mg ml^−1^ 2-PrOH solution at different angles and determined through Berry Plot **a**, and the hydrodynamic radius (*R*_h_) was calculated by measuring *D*_hθ_ of the sample solution at different angles by DLS and extrapolating the value **b**. Detailed calculation process was described in Methods part. **c** Plots of the solution temperature versus morphology factor *R*_g_/*R*_h_ (ρ) of formed PPV_12_-*b*-P2VP_22_ aggregations in 0.05 mg ml^−1^ 2-PrOH solution. **d** 2D GIWAXS pattern and **e** GIWAXS profiles along the in-plane direction of platelet 2-D square micelles obtained from PPV_12_-*b*-P2VP_22_ in 0.05 mg ml^−1^ 2-PrOH solution. The data cut in the plot was caused by the gap of the detector. **f** Molecular dynamics simulations of the assembly states in platelet 2-D square micelles of PPV_12_-*b*-P2VP_12_. The left one is the final self-assembled structure after simulation in isopropanol, and the solvent molecules are not shown for clarity. The right one is a local display of two neighborhood molecules in assembled structure. **g** Illustration of growing process of platelet 2-D square micelles of PPV_12_-*b*-P2VP_*n*_

Based on the changes in the UV-Vis spectra caused by changes in temperature and time, we could conclude that the molecular aggregation in solution was likely dependent on the temperature before cooling to room temperature. To characterize the aggregating state in solution during the cooling process, the 0.02 mg ml^−1^ 2-PrOH solution of PPV_12_-*b*-P2VP_22_ was examined by dynamic light scattering (DLS) and static light scattering (SLS), because molecules of PPV_12_-*b*-P2VP_22_ in 2-PrOH could completely assemble by H-aggregation without micelles stacking and concentration dependence. When we measured the solution by DLS above 50 °C, the scattering strength was too low to be detected, showing that the molecules in solution appeared dissolved with no particles large enough, which is consistent with the UV-Vis results. When the solution was cooled to 48 °C, features could be observed in the DLS detect and the hydrodynamic diameter (*D*_h_) of the aggregated micelle particles was determined to be 61.3 nm. As the solution was cooled to room temperature, the aggregated particle size quickly increased to 208.8 nm. Then, the rate of the increase in particle size slowed and the *D*_h_ changed from 208.8 nm to 275.7 nm after aging for 3.5 h (Supplementary Fig. 20). It is well known that the morphologies of particles can be revealed by their *R*_g_/*R*_h_ values (the morphology factor, *ρ*) in solution^32^. At a certain temperature, we calculated the *D*_hθ_ of the sample solution at different angles by DLS and extrapolated the value of *R*_h_, and the root mean square radius of gyration (*R*_g_) was calculated by measuring the SLS results of the copolymer solution at multiple angles less than 90°^33^. The *R*_g_ and *R*_h_ values of the self-assembled 2-D square micelles of PPV_12_-*b*-P2VP_22_ in the 2-PrOH solution after aged for 2 days were measured as 196.7 and 144.9 nm, respectively, and the *R*_g_/*R*_h_ value was calculated to be 1.36. According to previous reports^34,35^, the *R*_g_/*R*_h_ value of spherical micelles is close to 1, whereas that of coiled micelles and rigid rods are larger than 1.5. As we know, the researches of the *R*_g_/*R*_h_ value about 2-D materials, especially those with regular shapes, were rarely reported. The analogous particle system was regular stars whose value of *R*_g_/*R*_h_ was 1.33, which was surely close to the value of the formed 2-D square micelles. The geometric symmetry of 2-D platelet square micelles is higher than that of a coil but lower than that of a sphere, so it is reasonable that the *R*_g_/*R*_h_ value of these micelles is between 1 and 1.5. The *R*_g_/*R*_h_ values of particles assembled in solutions at 45, 40, 35, 30, and 25 °C were measured to be 2.59, 2.17, 2.00, 1.74, and 1.41, respectively (Fig. 6c, Supplementary Fig. 21, and Table 3). It could be concluded that the originally formed aggregates of PPV_12_-*b*-P2VP_22_ at around 45 °C were one-dimensional, because the *R*_g_/*R*_h_ value was much larger than 2. As the temperature of the solution decreased, the *R*_g_/*R*_h_ value of the aggregates in solution gradually decreased until it reached ~1.36, which was the morphology factor (*ρ*) of 2-D platelet square micelles. The trend in the change in the *R*_g_/*R*_h_ values at different temperatures revealed that the molecules of PPV_12_-*b*-P2VP_*n*_ initially assembled into 1-D aggregates in the solution during the cooling process, gradually grew into 2-D square micelles as the solution cooled to room temperature, and finally the scales of the 2-D square micelles increased slowly during the aging period^35^.

### Molecular packing of platelet 2-D square micelles

We used diblock copolymers of PPV_12_-*b*-P2VP_*n*_ to achieve regular and homogeneous supramolecular 2-D fluorescent square micelles by dissolving–cooling–aging process. The scales of the achieved 2-D square micelles were controlled by the block ratio of PPV/P2VP and the concentration of the solution. In previous reports, the OPV copolymer with linear alkyl chains can be used to prepare monodisperse fiber-like micelles^22–24^. Therefore, we inferred that the formation of 2-D morphology is caused by the unique molecular packing related to the branched alkyl side chains. As discussed above, the conjugated PPV_12_ blocks should be arranged edge-on in the 2-D square micelles, which was confirmed by AFM measurements and DFT calculations.

To reveal the details of the molecular arrangements, appropriate characterization techniques for 2-D square micelles were necessary. First, we hoped to have the aid of selected-area electron diffraction (SAED). However, the SAED pattern indicated the formed 2-D square micelles were almost non-crystalline (Supplementary Fig. 22a). High-resolution TEM (HRTEM) photographs also confirmed the non-crystalline morphology of the 2-D square micelles, as there was no clear crystal lattice observed on the surface of the square micelle (Supplementary Fig. 22b).

Then, 2-D square micelles of PPV_12_-*b*-P2VP_22_ and PPV_12_-*b*-P2VP_36_ were characterized by grazing incidence wide-angle X-ray scattering (GIWAXS) to investigate the molecular packing in the assembly structures (Fig. 6d and Supplementary Fig. 25a). The 2-D square micelles of the two BCPs exhibited similar 2-D GIWAXS patterns. From the profiles along the in-plane direction (Fig. 6e), the reflections (100) and (200) were clearly visible, and no periodic reflection peaks along *q*_*z*_ were observed. This suggested that PPV_12_-*b*-P2VP_22_ and PPV_12_-*b*-P2VP_36_ molecules favor an edge-on configuration in the 2-D platelet square micelles, which is consistent with previously discussed data. The *d* spacing of PPV_12_-*b*-P2VP_22_ molecules arranged in the 2-D square micelles was calculated as 9.46 Å. The reflection peak, *q* = 1.505 Å^−1^, could be attributed to the (010) reflection, and the *d* spacing of 3.70 Å, calculated from peak *q* = 1.699 Å^−1^, originates from the π–π stacking of the PPV_12_ backbones parallel to the substrate. The angle of the adjacent molecular chain was 135° (Supplementary Fig. 26). A similar result could also be obtained from GIWAXS measurement of PPV_12_-*b*-P2VP_36_. According to the previous literatures^36,37^, the PPV molecules without side chains should adopt a herringbone molecular arrangement, where molecules are packed in layer-by-layer mode and within one layer the adjacent molecules are parallel to each other. This herringbone molecular arrangement agreed with the data measured by our GIWAXS experiments, indicating that the molecules of PPV_12_-*b*-P2VP_*n*_ in the 2-D platelet square micelles should pack through π–π interactions and probably adopt a herringbone configuration. This molecular arrangement was also supported by molecular dynamics simulations (Fig. 6f). From the simulation results, the PPV_12_ blocks and the P2VP blocks aggregated together respectively because of the hydrophilic–hydrophobic interactions. The PPV_12_ blocks interlaced with each other, and the adjacent π-conjugated PPV_12_ blocks arranged to form a V shape with the side alkyl chains inserted between them. In the diagonal direction, the molecules had the same advantage of growing orientation, which possibly caused the formation of the square shapes of the 2-D micelles^38^.

As illustrated in Fig. 6g, the process by which the platelet 2-D square micelles form could be determined. Initially, at 85 °C, the molecules of PPV_12_-*b*-P2VP_*n*_ are completely dissolved in 2-PrOH and move freely in solution. As the temperature of the solution decreased, the quickly moving molecules started to aggregate driven by the hydrophobic interaction producing close distances for the PPV blocks. Then, the initially formed aggregates should be 1-D polymer micelles derived from π-π intermolecular interactions of the PPV_12_ cores, and these architectures gradually grow into 2-D platelet square micelles because of intermolecular π-π stacking of the rigid conjugated PPV_12_ cores from two directions. It was observed that the branched alkyl chains of the PPV_12_ cores played a crucial role in the formation of the platelet 2-D square micelles. First, the branched alkyl chains improved the solubility of the PPV_12_ cores in 2-PrOH, changed the hydrophilic-hydrophobic interactions and slowed the aggregation rate of PPV_12_-*b*-P2VP_*n*_ BCPs. More importantly, it was the existence of the branched alkyl chains that led to the herringbone molecular arrangement, which induced the formation of the square micelles.

### Platelet 2-D square micelles as carrier of SiO_2_ NPs

As described above, the P2VP coronas were distributed on the top and bottom surfaces of the platelet 2-D square micelles. It is known that SiO_2_ nanoparticles (NPs) can combine with P2VP coronas through hydrogen bonding^39^. Therefore, we hoped the formed 2-D square micelles of PPV_12_-*b*-P2VP_*n*_ could be functionalized carrier templates of SiO_2_ NPs. We selected 2-D square micelles originating from PPV_12_-*b*-P2VP_22_ and mixed them with SiO_2_ NPs (diameter = 20 nm) in ethanol. After aging for 1 day, all the platelet 2-D square micelles were loaded with SiO_2_ NPs. The sizes of the patterns of SiO_2_ NPs loading on square micelles were consistent with the scales of the square platelets (Supplementary Fig. 27). The SiO_2_ NPs were more concentrated on the platelet edges perhaps because the steric encumbrance at the boundary was lower^40^.

## Discussion

In summary, we have described a simple strategy for the preparation of uniform, colloidally stable, 2-D platelet square micro- and nano-structures of PPV_12_-*b*-P2VP_*n*_ BCPs through intermolecular π–π stacking interactions and hydrophobic interactions. The size and thickness of the formed 2-D square micelles could be effectively controlled by the ratio of PPV/P2VP blocks and they exhibited different concentration dependences. The square micelles initially form 1-D structures in 2-PrOH and then grow into 2-D architectures, and the growth is related to temperature and aging time. The formation of the square shapes is likely induced by the herringbone molecular arrangement, which is caused by the branched alkyl chains attached to the conjugated PPV_12_ cores. This strategy is practical and expected to be easily applicable to other conjugated block based copolymer systems. Moreover, these 2-D square film micelles possess high stability and flexibility, large area-to-volume ratios and definite fluorescence storage, which have potential applications in the fields of biosensors, catalysis, template building, and optoelectronics on the nanoscale.

## Methods

### Equipment and materials

The BCP poly(2,5-di(2′-ethylhexyloxy)-1,4-phenylenevinylene)12-aldehyde (PPV_12_-CHO) homopolymer was prepared by literature procedures^33^, and the fluorescent diblock copolymers PPV_12_-*b*-P2VP_*n*_ used for building blocks of self-assembly were synthesized by quenching the anionic polymerization of P2VP with the PPV_12_-CHO^41^. The detailed procedures were described in Supplementary Methods. The anionic polymerization was carried out in a glove box filled with nitrogen atmosphere. The block ratios of BCPs were determined by GPC and ^1^H NMR spectroscopy. GPC measurements were carried out on a Viscotek VE 2001 triple-detector gel permeation chromatograph equipped with an automatic sampler, a pump, an injector, an inline degasser, and a column oven (40 °C). The elution columns consist of styrene/divinyl benzene gels with pore sizes of 500 and 100,000 Å. THF was used as the eluent, with a flow rate of 1.0 ml min^−1^. Samples were dissolved in the eluent (1 mg ml^−1^) and filtered with an organic phase filter (polytetrafluoroethylene membrane of 0.45 mm pore size) before analysis. The calibration was conducted using a PolyCALTM polystyrene standard from Viscotek. ^1^H NMR spectra were measured on a Bruker AVANCE 400 MHz spectrometer with tetramethylsilane as the internal standard.

### Preparation of platelet 2-D square micelles

A 0.01 mg ml^−1^ solution of PPV_12_-*b*-P2VP_12_ or PPV_12_-*b*-P2VP_16_ with spectrum pure 2-PrOH was prepared in a 20 ml sealed vial, and for PPV_12_-*b*-P2VP_*n*_ with lower ratio of PPV/P2VP blocks, the initial concentration of prepared solution was commonly 0.05 mg ml^−1^. Then the first prepared concentrated solution was diluted to a certain concentration. The prepared solution was heated at 85 °C for 45 min and then the pale yellow solution was slowly cooled to room temperature (25 °C) and aged for 24 h. The formed platelet 2-D square micelles were observed by TEM analysis of the drop-cast solution.

### Transmission electron microscopy

TEM samples were prepared by drop-casting 5 μl of the micelle-formed solution onto a carbon-coated copper grid followed by solvent evaporated. TEM micrographs were obtained on a Hitachi HT7700 microscope operating at 80 kV and equipped with an AMF-5016 charge-coupled device camera. For the statistical analysis, the obtained images were analyzed by using DigitalMicrograph software package developed by the US Gatan company. The diagonal length was calculated to characterize the scale of the platelet 2-D square micelles. Two hundred 2-D square micelles from TEM images were traced by hand. *D*_n_ (number-average diagonal length) and *D*_w_ (weight-average diagonal length) of each sample of platelet 2-D square micelles were determined from these data by using Eqs (1) and (2), respectively (where *D*_*i*_ is the diagonal length of individual platelet 2-D square micelles, *N*_*i*_ is the number of diagonal length *D*_*i*_).

1 $$D_{\mathrm{n}} = \frac{{\mathop {\sum }\nolimits_{{ i} = 1}^N N_{ i}D_{ i}}}{{\mathop {\sum }\nolimits_{{ i} = 1}^N N_{ i}}}$$

2 $$D_{\mathrm{w}} = \frac{{\mathop {\sum }\nolimits_{{ i} = 1}^N N_{ i}D_{ i}^2}}{{\mathop {\sum }\nolimits_{{ i} = 1}^N N_{ i}D_{ i}}}$$

HRTEM photographs were obtained on a Tecnai F30 microscope operating at 120 kV. Used TEM samples were prepared by drop-casting 5 μl of the micelle-formed solution onto a micro copper grid followed by solvent evaporated. SAED patterns for 2-D platelet square micelles were also collected by Tecnai F30 TEM.

### Atomic force microscopy

The AFM samples for detecting were prepared by drop-coating about 15 μl of the 2-D square micelle solution onto a pre-cleaned and treated silicon wafer and evaporating the solvent 2-PrOH. The silicon wafers used for substrate were cleaned in piranha solution for 30 min, then ultrasound treated successively in ethanol, water, ultrapure water, and finally dried with blowing nitrogen. The images were obtained using an Asylum Research AFM in AC mode under ambient conditions. The used sensor cantilevers was Silicon probe reflex coated with aluminum manufactured by Budgetsensors Company. Images were analyzed using IGOR Pro software developed by WaveMetrics Inc.

### Theoretical calculations

The ground state geometries were fully optimized by the DFT method with the Becke three-parameter hybrid exchange and the Lee–Yang–Parr correlation functional (B3LYP) and 6–31G* basis set using the Gaussian 09 software package.

### Scanning electron microscopy

Samples for SEM were prepared by drop-casting about 15 μl of the 2-D square micelle solution onto a pre-cleaned and treated silicon wafer and evaporating the solvent 2-PrOH. The silicon wafer was treated the same with AFM substrates as described above. Imaging was performed using a Zeiss Merlin SEM microscope at 5 kV accelerate voltage and 100 pA beam intensity.

### Laser scanning confocal microscopy

LSCM images were performed using a Leica SP8 confocal attached to a Leica DMI6000 inverted epifluorescence microscope with a × 63 (numerical aperture1.4) oil-immersion objective lens. A drop of sample solution of micelles was put on a cleaned slide followed sealing with a cover slip, which was prepared for observing. The samples of formed 2-D square micelles in solution were excited using an argon laser operating at 405 nm and the confocal images were obtained using digital detectors with observation windows of 530–630 nm. The Supplementary Movie 1 was obtained by collecting three frames of confocal images per second.

### UV-Vis absorption spectra

The UV-Vis absorption spectra were recorded by an Agilent Cary 60 UV-Vis spectrophotometer. A capped cuvette filled with 2-PrOH solution of PPV_12_-*b*-P2VP_*n*_ was placed in the holder of spectrophotometer that was linked with a temperature controller. The solution in cuvette was heated to 85 °C and kept the temperature for 45 min. Then the heating was stopped, and the solution was spontaneous cooled to room temperature. The UV-Vis spectrum and the real-time temperature of the solution was recorded every 3 min during cooling down process. After the sample solution was cooled to room temperature, the measurement was changed to be carried out every 30 min until after aging for 20 h (for the 0.01 mg ml^−1^ 2-PrOH solution of PPV_12_-*b*-P2VP_36_, the measurement lasted to 72 h).

### PL spectra and fluorescence quantum efficiency

The PL spectra were recorded by an Edinburgh FS5 spectrofluorometer and the fluorescence quantum efficiencies were detected on a Hamamatsu C11347 absolute PL quantum yield spectrometer. The samples were prepared by dissolving PPV_12_-*b*-P2VP_*n*_ in THF and 2-PrOH under concentration of 0.01 mg ml^−1^ and the prepared 2-PrOH solutions were heated at 85 °C for 45 min, cooled to room temperature (25 °C), and aged for 24 h.

### DLS and SLS measurements

DLS and SLS measurements were performed using a Brookhaven BI-200SM wide-angle laser light-scattering apparatus equipped with a 635 nm red laser. PPV_12_-*b*-P2VP_22_ solution of 0.05 mg ml^−1^ in 2-PrOH was placed in sealed glass cuvette, and the cuvette was placed in pool full of matching fluids whose temperature was controlled by a water cycle system. The solution in cuvette was heated at 85 °C for 45 min and then spontaneously cooled to room temperature with DLS measurement carried out every 5 min. After sample solution was cooled to room temperature, the DLS was measured every 10 min until aging for 2 h, and then the measurement interval was changed to 1 h. The morphology factor *ρ* was calculated by the ratio of *R*_g_/*R*_h_. *R*_h_ represents the hydrodynamic radius and *R*_g_ represents the root mean square radius of gyration. *R*_h_ was calculated with the method of measuring *D*_h_ of the sample solution under different angles by DLS. PPV_12_-*b*-P2VP_22_ solution of 0.05 mg ml^−1^ in 2-PrOH placed in sealed glass cuvette was heated at 85 °C for 45 min and then it was cooled to a certain temperature. Under the temperature, hydrodynamic diameter of particles in sample solution was measured at 30°, 45°, 60°, 75°, and 90°, respectively. Drew a plot of *q*^2^ versus *D*_h*θ*_, extrapolated the value of *D*_h0_ by least squares curve fitting method and obtained the value of *R*_h_. At the same temperature, SLS was measured under 30°, 45°, 60°, 75°, and 90°, respectively. *R*_g_ is consistent with Eq. (3). As the size of particles at different concentrations does not vary greatly, the Eq. (3) can be simplified to Eq. (4) (*I* stand for scattering intensity; *q* represent for scattering vector, and it can be calculated by Eq. (5); in Eq. (5), *n* is refractive index of solution, *λ* is laser wavelength, and *θ* is measuring angle). Then the value of *R*_g_ can be calculated from primary function Eq. (4).

3 $$\frac{{KC}}{I} = \frac{1}{{M_{\mathrm{w}}}}\left( {1 + \frac{{16\pi ^2}}{{3\lambda ^2}}{\mathrm{sin}}^2\left( {\frac{\theta }{2}} \right)R_{\mathrm{g}}^2} \right) + 2A_2C$$

4 $$\frac{{k_1}}{I} = k_2 + \frac{1}{3}q^2R_{\mathrm{g}}^2$$

5 $$q = \frac{{4\pi n\,{\mathrm{sin}}\left( {\frac{\theta }{2}} \right)}}{\lambda }$$

### Wide-angle X-ray diffraction

The wide-angle X-ray diffraction (WXRD) samples for detecting were prepared by drop-coating about 15 μl of the 2-D square micelle solution onto a pre-cleaned and treated silicon wafer and evaporating the solvent 2-PrOH for 10 times. The silicon wafer was treated the same with AFM substrates as described above. The WXRD spectrum was recorded using a Rigaku Smartlab 9 kW X-ray diffractometer using CuKα radiation (*λ* = 1.5418 Å).

### GIWAXS measurements

The GIWAXS samples for detecting were prepared by drop-coating about 15 μl of the 2-D square micelle solution onto a pre-cleaned and treated silicon wafer and evaporating the solvent 2-PrOH for five times. The silicon wafer was treated the same with AFM substrates as described above. GIWAXS measurements were performed at the 8ID-E beamline at the Advanced Photon Source, Argonne National Laboratory, using X-rays with a wavelength of *λ* = 1.1385 Å, and a beam size of 200 μm (h) and 20 μm (v). A 2-D PILATUS 1M-F detector was used to capture the scattering patterns and was situated at 208.7 mm from samples. Typical GIWAXS patterns were taken at an incidence angle of 0.13°, above the critical angles of conjugated polymer and below the critical angle of the silicon substrate. Consequently, the entire structure could be detected. In addition, the *q*_*y*_ scan was obtained from a linecut across the reflection beam center, while the *q*_*z*_ scan was achieved by a linecut at *q*_*y*_ = 0 Å^−1^, using the reflected beam center as 0.

### Molecular dynamics simulation methods and details

First, we built a single PPV_12_-*b*-P2VP_12_ molecule by using Material Studio software package developed by the US Accelrys company and we optimized the configuration of this molecule with a steep descent energy minimization step. Thereafter, we packed 12 molecules on a 4 × 3 lattice with large enough intermolecular distances to avoid the initial direct contact between these molecules. All molecules were aligned parallel to the *z* direction of the simulation box. Periodic boundary conditions were used in our simulation. The simulation box had an initial box size of 18 nm in *x* and *y* direction, and 20 nm in the *z*-direction. This initial bundle of 12 molecules was put in the center of the simulation box. Afterward, the isopropanol solvent molecules were added into the simulation box at random positions. The initial mass density of the initial configuration was 0.2 g cm^−3^. From the initial configuration, we performed full atomistic MD simulations by using GROMACS 5.1 software package, all simulations were performed by applying hybrid/heterogeneous acceleration paralleled on two central processing units (CPU) (Intel(R) Xeon(R) CPU E5-2673 V3) and four graphics processing units (GPU) (NVIDIA 1080). Through steep descent energy minimization, the initial configuration was locally equilibrated. Furthermore, a designed NPT run of 10 ns is performed for equilibration of the system density. The final mass density of the system was 0.814 g cm^−3^. After which, the system had a size of 10.94 nm in *x* and *y* direction, and 16.73 nm in *z* direction. Afterward, we performed the production run for another 50 ns in NVT (N represents atomic number in system, V represents volume and T represents temperature) ensemble. In the initial equilibration run, the Berendsen thermostat and barostat were used with a thermostat coupling time of 0.2 ps, and a barstat coupling time of 1 ps, respectively. However, in the final NVT production run the Nosé–Hoover thermostat was used. The optimized potentials for liquid simulations-all atom (OPLS-AA) force field parameters were used in our simulation. The simulation temperature *T* was set at 298 K. The particle-mesh Ewald method was used to treat the electrostatic interactions in the system. The leapfrog algorithm is employed to integrate Newtons’ equations of motion with a time step of 1 fs.

### Data availability

The data that support the findings of this study, including the Supplementary Information, are available from the corresponding author on request.

## Electronic supplementary material

Supplementary Information

Description of Additional Supplementary Files

Supplementary Movie 1
