# Supplementary material for: Uniform two-dimensional square assemblies from conjugated block copolymers driven by π–π interactions with controllable sizes
Source: Nat Commun. 2018 Feb 28;9:865. doi: 10.1038/s41467-018-03195-y (PMC5830438; doi:10.1038/s41467-018-03195-y)
Supplement: Supplementary file 1 — Supplementary Information [file 41467_2018_3195_MOESM1_ESM.pdf]

Supplementary Information

Uniform Two-dimensional Square Assemblies from Conjugated Block Copolymers Driven by π-π Interactions with Controllable Sizes

Feng He* *et al.*

Supplementary Methods

Polymer Synthesis and Characterization. All of air- or moisture-sensitive reactions were carried out in dried glassware under an argon pressure using Schlenk techniques or in a glove box under nitrogen atmosphere. The detailed procedure path of synthesis was illustrated in Supplementary Figure 1.

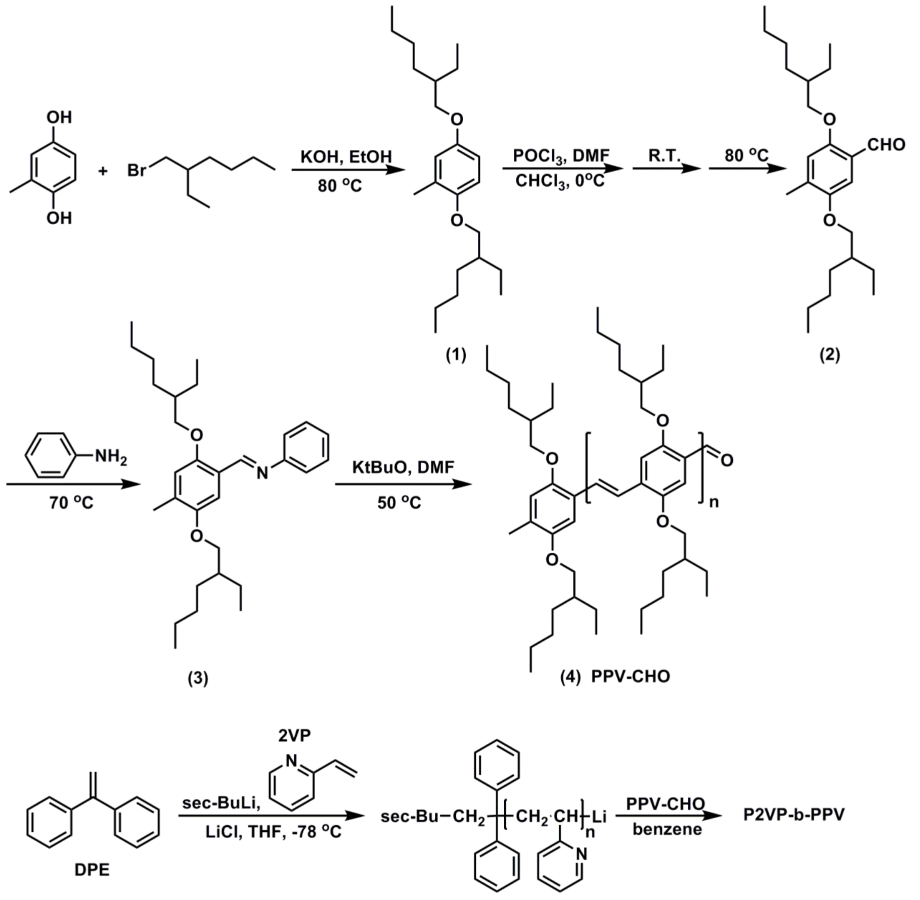

Supplementary Figure 1. Schematic synthesis route for diblock copolymers PPV-*b*-P2VP.

Synthesis of 1,4-bis(2-ethylhexyloxy)-2-methylbenzene (1). A mixture of 2-methylbenzene-1,4-diol (12.4 g, 0.1 mol) and KOH (16.42 g, 0.29 mol) in ethanol (170 mL) was refluxed at 80 °C for 1 h under Ar atmosphere. Then 3-(bromomethyl)heptane (52 mL, 0.29 mol) was added dropwise to the stirring refluxing solution. The mixture was refluxed overnight. After cooling to room temperature, 700 mL water was added into mixture. The mixture was extracted with anhydrous ether (3 × 100 mL), then the gathered organic phase was dried with $MgSO_4$, finally the solvent was removed by rotary evaporator. After purification by chromatography (silica gel, CH_2Cl_2/ petroleum ether 1:9), a pale yellow oil (**1**, 26.13 g, 0.075 mol, 74.99%) was obtained. ^1H NMR (400 MHz, CDCl3): δ 6.75-6.70 (m, 2H), 6.65 (dd, 1H), 3.80-3.75 (m, 4H), 2.20 (s, 3H), 1.73-1.65 (m, 2H), 1.55-1.36 (m, 8H), 1.36-1.24 (br, 8H), 0.92 (t, 12H).

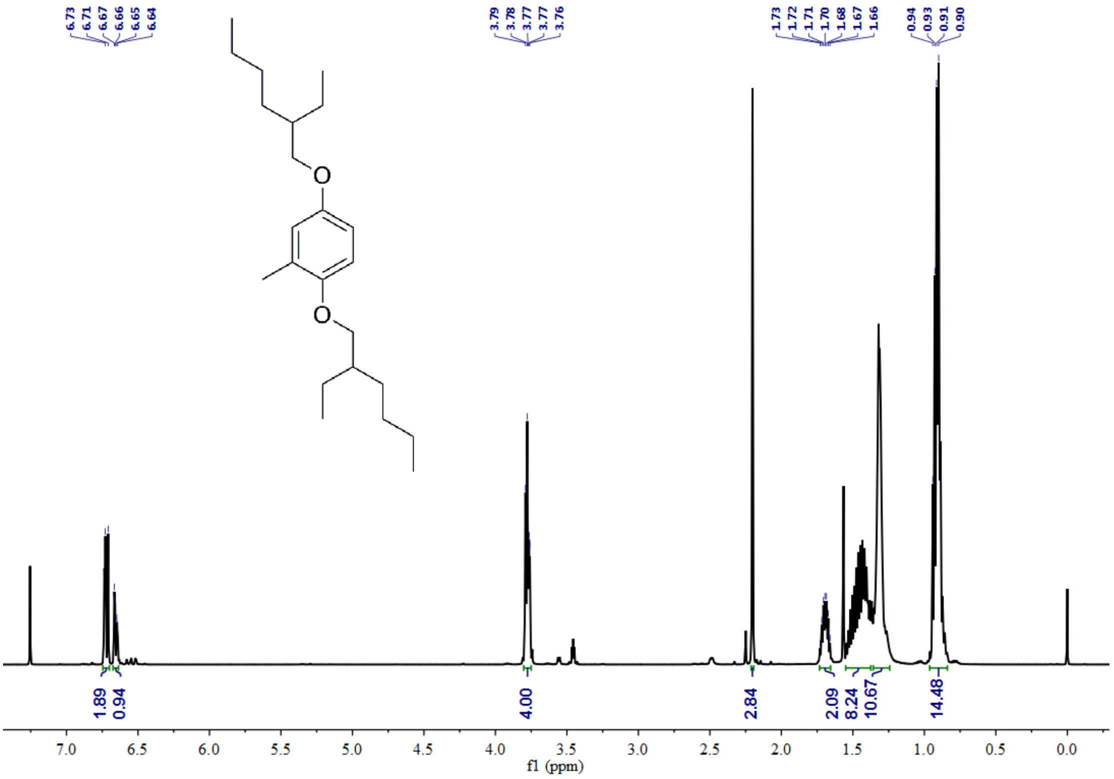

Supplementary Figure 2. ^1H NMR spectra of 1,4-bis(2-ethylhexyloxy)-2-methylbenzene in $CDCl_3$.

Synthesis of 2,5-bis(2-ethylhexyloxy)-4-methylbenzaldehyde (2). N, N-dimethylformamide (DMF, 17 mL) and phosphorus oxychloride (24 mL) was successively added dropwise into stirring solution of 1,4-bis(2-ethylhexyloxy)-2-methylbenzene (compound **1**, 20.9 g, 60 mmol) in 30 mL chloroform at 0 °C. After continuing stirring for 1 h at room temperature, the mixture was refluxed at 80 °C for 48 h followed by adding 100 mL ice water to stop reaction. The mixture was extracted by dichloromethane (3 × 50 mL), and then the organic phase was washed with aqueous $NaHCO_3$. After dried by $MgSO_4$ and rotary evaporating, the obtained crude oil was purified by chromatography (silica gel, CH_2Cl_2/ petroleum ether 1:1), a yellow oil (**2**, 16.27 g, 43.20 mmol, 72.06%) was got. ^{1}H NMR (400 MHz, $CDCl_3$): δ 10.43 (s, 1H), 7.23 (s, 1H), 6.80 (s, 1H), 3.92 (d, 2H), 3.84 (d, 2H), 2.28 (s, 3H), 1.80-1.69 (m, 2H), 1.55-1.38 (m, 8H), 1.37-1.25 (br, 8H), 0.97-0.87 (m, 12H).

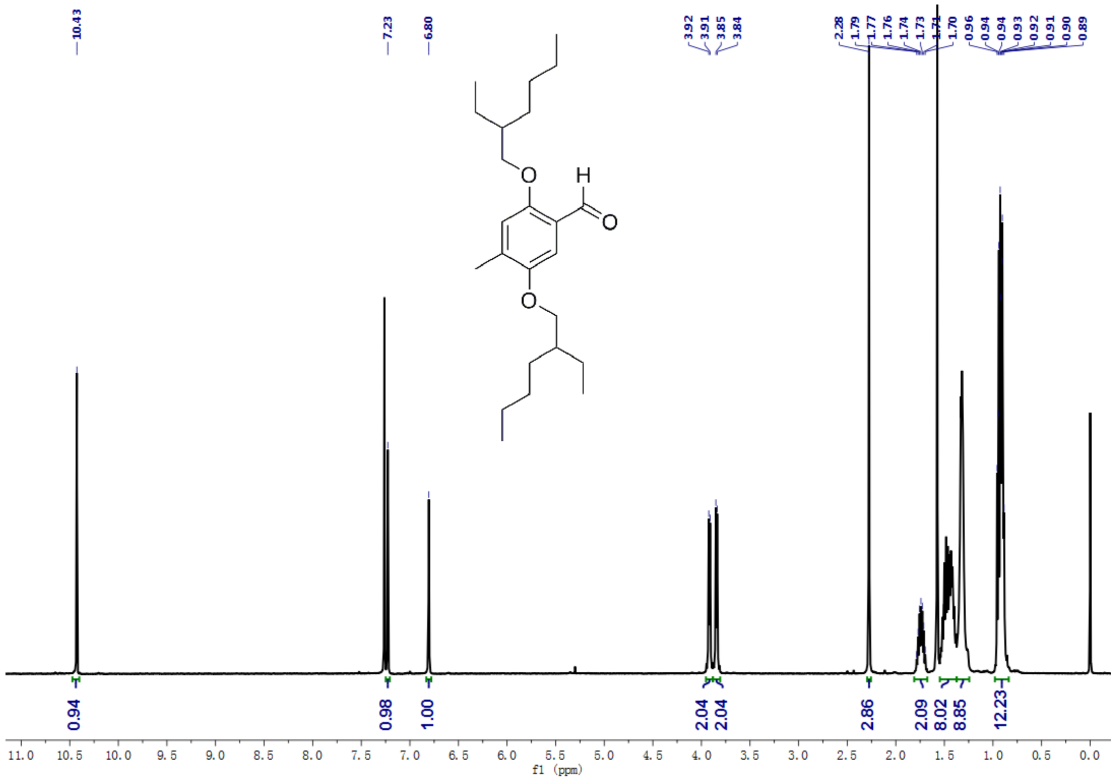

Supplementary Figure 3. ^{1}H NMR spectra of 2,5-bis(2-ethylhexyloxy)-4-methylbenzaldehyde in $CDCl_3$.

Synthesis of N-(2,5-bis(2-ethylhexyloxy)-4-methylbenzylidene)aniline (3). A mixture of 2,5-bis(2-ethylhexyloxy)-4-methylbenzaldehyde (compound **2**, 5.7 g, 15.15 mmol) and aniline (3.08 g, 33.03 mmol) was stirred for 2h at 70 °C under reduced pressure, then the temperature of the mixture raised to 110 °C to remove excess aniline. The yielded deep yellow viscous oil (3, 6.56 g, 14.53 mmol, 95.91%) needed to be stored at low temperature and under dark cause it was easy to decompose. ^{1}H NMR (400 MHz, $CDCl_3$): δ 8.87 (s, 1H), 7.57 (s, 1H), 7.42-7.35 (m, 2H), 7.23-7.19 (m, 3H), 6.78 (s, 1H), 3.93 (d, 2H), 3.89 (d, 2H), 2.28 (s, 3H), 1.80-1.69 (m, 2H), 1.55-1.38 (m, 8H), 1.37-1.24 (br, 8H), 0.97-0.85 (m, 12H).

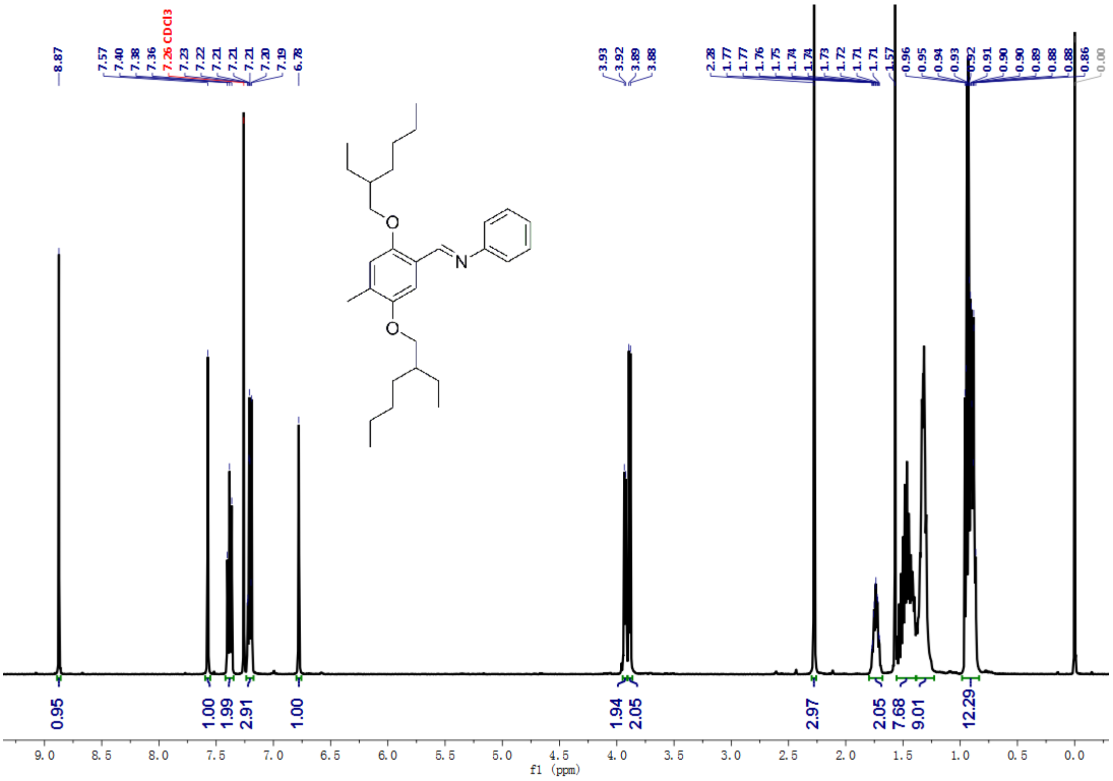

Supplementary Figure 4. ^{1}H NMR spectra of N-(2,5-bis(2-ethylhexyloxy)-4-methylbenzylidene) aniline in $CDCl_3$.

Synthesis of Poly(2,5-di(2'-ethylhexyloxy)-1,4-phenylenevinylene)-aldehyde (4, PPV-CHO). In a round bottom flask, potassium tert-butoxide (1.12 g, 9.97 mmol) was stirred in 120 mL anhydrous DMF at 50 °C. Maintaining temperature, a solution of N-(2,5-bis(2-ethylhexyloxy)-4-methylbenzylidene)aniline (compound **3**, 1.5 g, 3.32 mmol) in 20 mL anhydrous DMF was added into mixture. After stirring for 1h at the same temperature, the mixture was poured into 250 mL 1M hydrochloric acid and stirred for 48 h. Then the reaction mixture was extracted with chloroform (3 × 50 mL), and the organic phase was successively washed by aqueous $NaHCO_3$ and water followed by dried with $MgSO_4$ and rotary evaporating. Product in low dispersity was successively collected and fractionated by methanol, acetone and hexane with Soxhlet extraction, and the fractionated product in hexane was precipitated into methanol. The value of Mn = 3800 and the number-average degree of polymerization n = 12 for PPV-CHO was determined by ^{1}H NMR. The polydispersity index was measured at 1.045 by GPC in THF. ^{1}H NMR (400 MHz, $CDCl_3$): δ 10.46 (s, 1H), 7.69-7.42 (m), 7.35-7.10 (m), 6.73 (s, 1H), 4.09-3.81 (m, 48H), 2.24 (s, 3H), 2.05-1.98 (m), 1.70-1.43 (m), 1.43-1.29 (m), 1.28-1.23 (m), 1.04-0.95 (m), 0.95-0.86 (m).

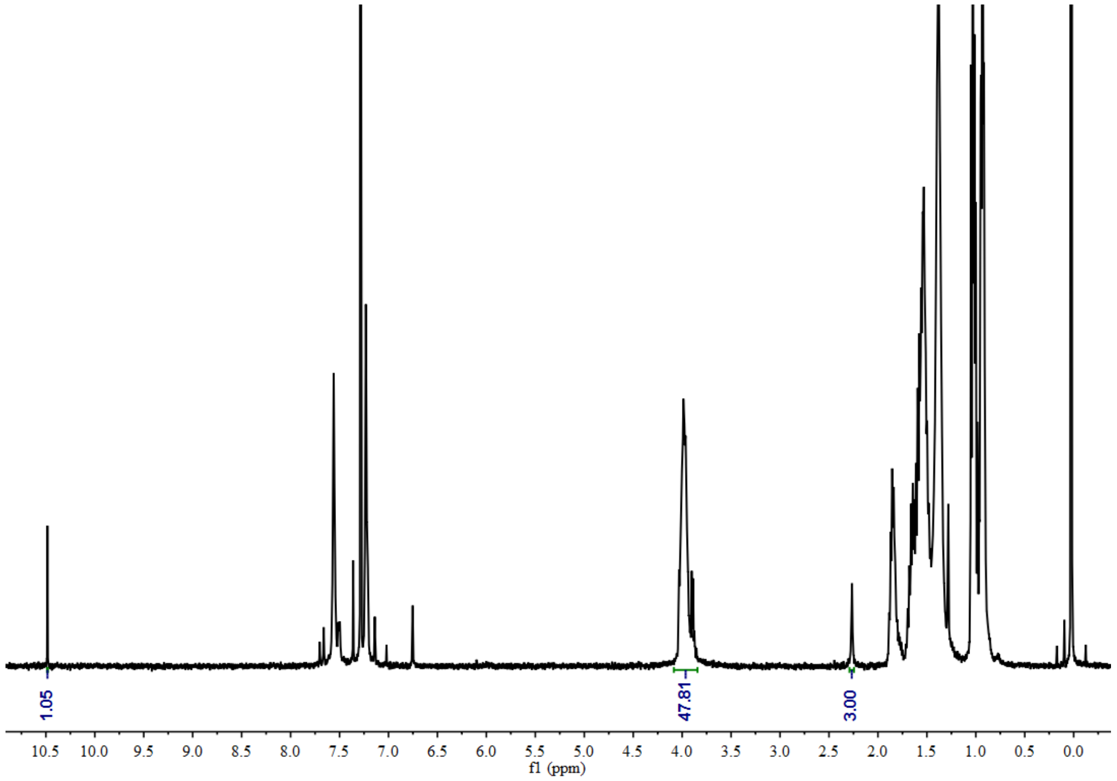

Supplementary Figure 5. ^{1}H NMR spectra of PPV_{12}-CHO in $CDCl_3$.

Synthesis of Poly(2,5-di(2'-ethylhexyloxy)-1,4-phenylenevinylene)-block-poly (2-vinylpyridine) (5, PPV-*b*-P2VP). In a glovebox filled with N_2 at -78 °C, 86.9 μL of sec-BuLi (1.3 M in hexane) was quickly added to a stirring solution of dried LiCl (45.7 mg, 1.13 mmol) and 1,1-diphenylethylene (DPE, 20 μL, 0.113 mmol) in THF (2 mL). Then maintaining -78 °C, a certain amount of 2-vinylpyridine (2VP) in THF (2 mL) was added to the mixture. After the reaction went on running for 2 h at -78 °C, a solution of PPV_{12}-CHO (43 mg, 0.01 mmol) in benzene (2 mL) was quickly added into the reaction mixture to quench the anion polymerization. Then the reaction temperature was increased to room temperature and stirred for additional 12 h. As the reaction stopped, the reaction mixture was poured into 25 mL chloroform and washed successively with aqueous HCl (pH = 3), water and aqueous $NaHCO_3$. After rotary evaporating, the crude residue was purified by chromatography (silica gel, eluent was changed from chloroform to chloroform/Et_3N 9:1), and finally red solid was obtained. The block ratio of produced PPV_{12}-*b*-$P2VP_n$ was determined by ^{1}H NMR and GPC.

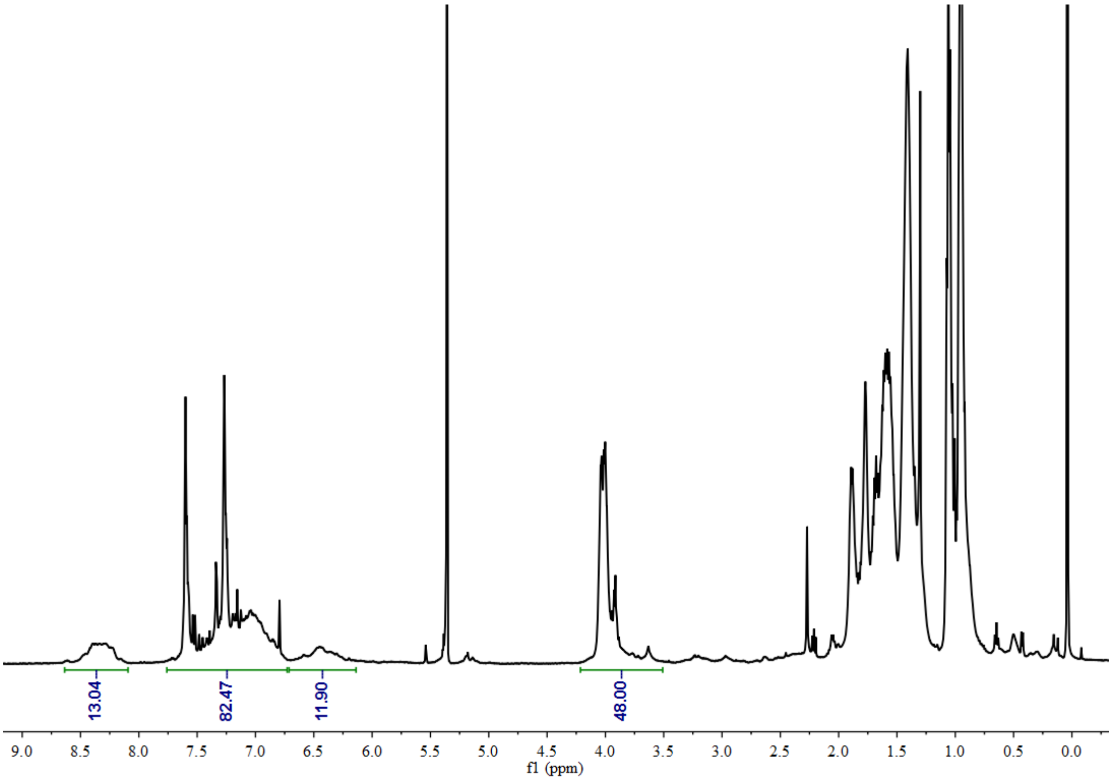

Supplementary Figure 6. ^{1}H NMR spectra of PPV_{12}-*b*-$P2VP_{12}$ in CD_2Cl_2.

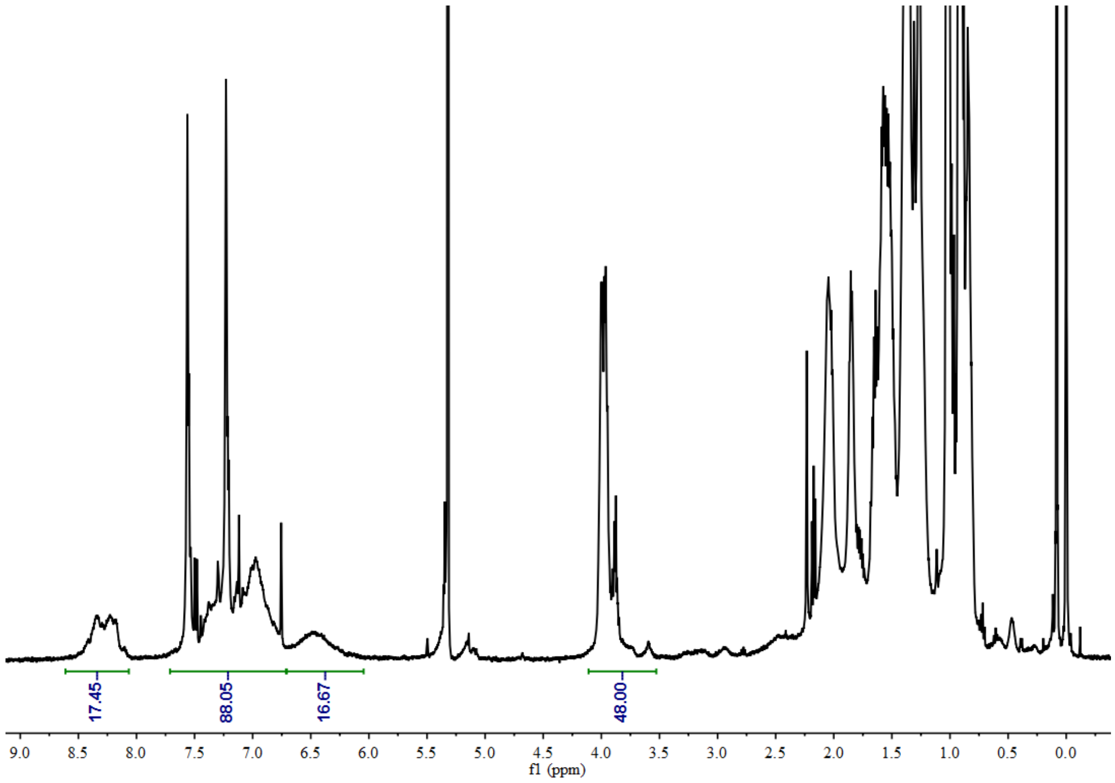

Supplementary Figure 7. ^{1}H NMR spectra of PPV_{12}-*b*-$P2VP_{16}$ in CD_2Cl_2.

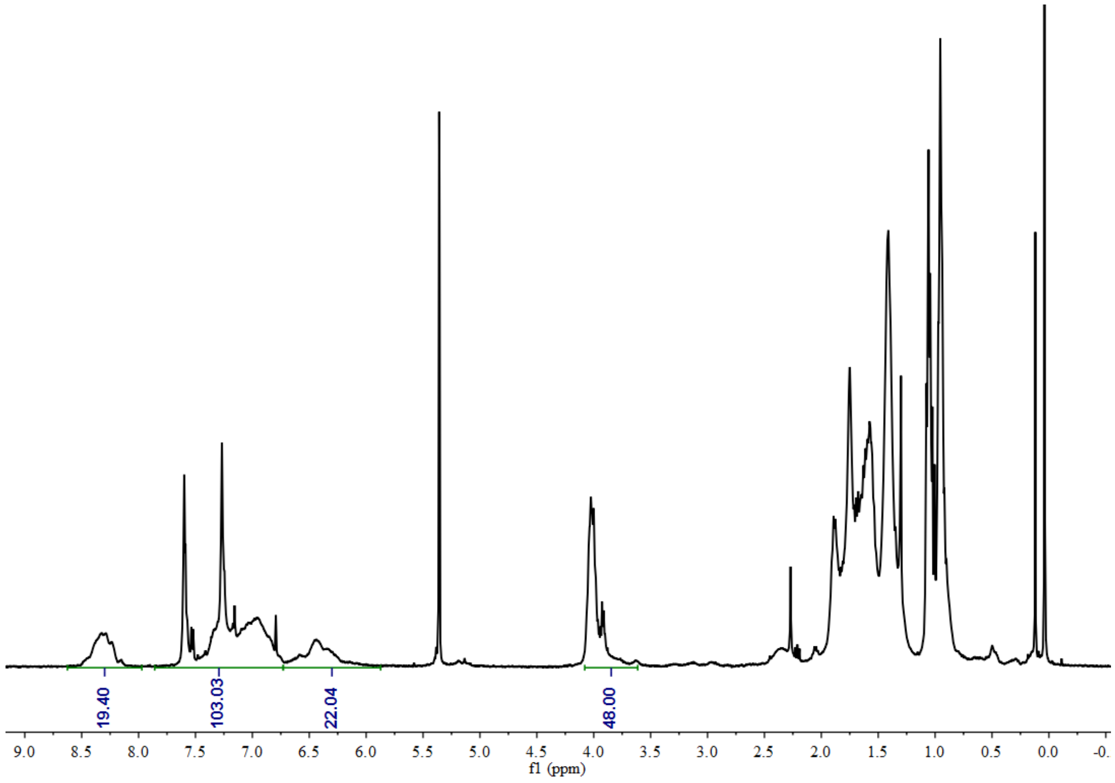

Supplementary Figure 8. ^{1}H NMR spectra of PPV_{12}-*b*-$P2VP_{22}$ in CD_2Cl_2.

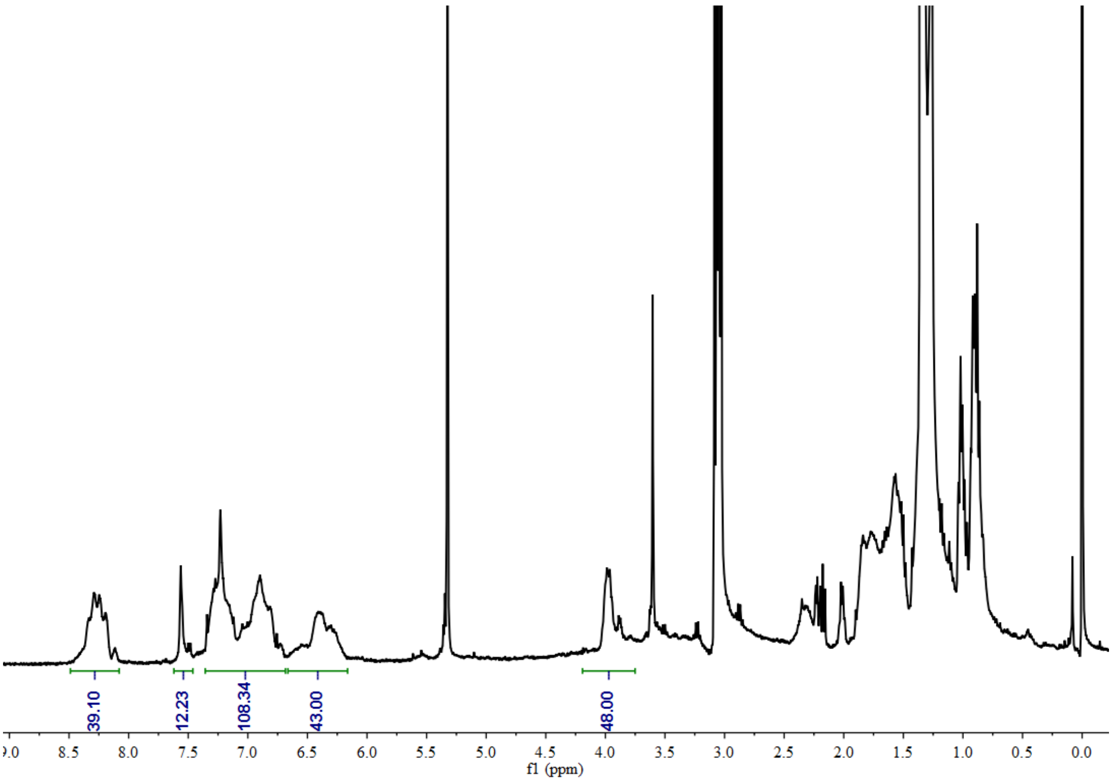

Supplementary Figure 9. ^{1}H NMR spectra of PPV_{12}-*b*-$P2VP_{36}$ in CD_2Cl_2.

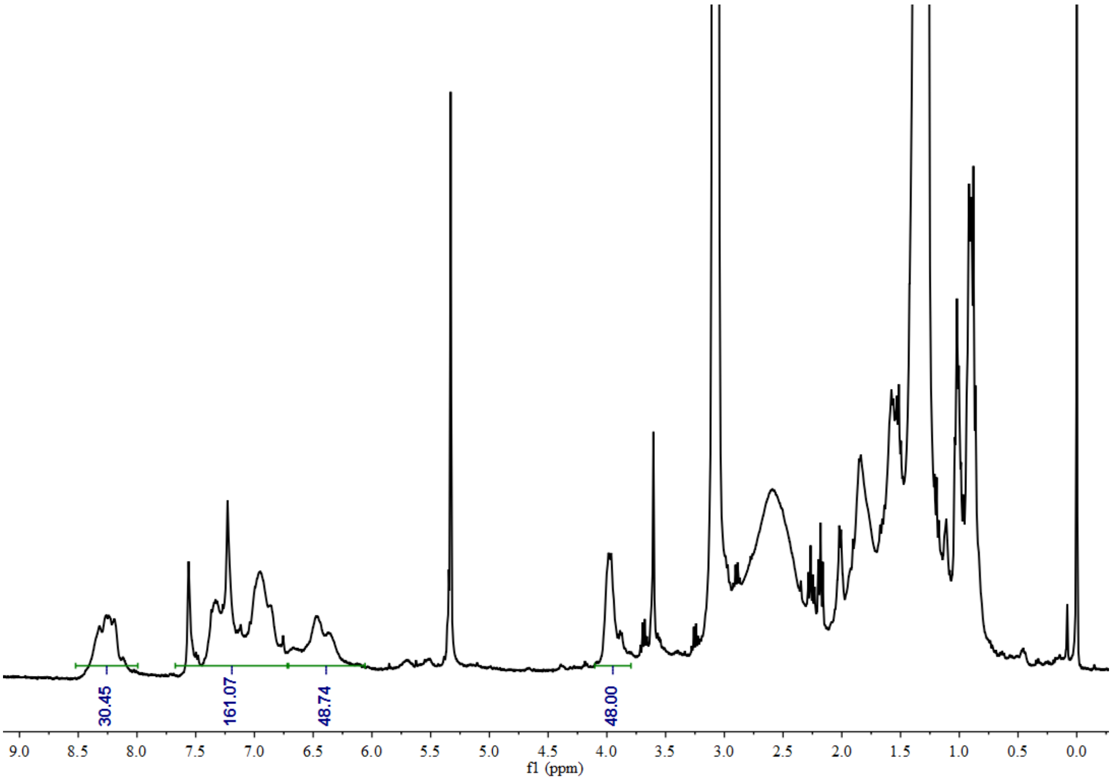

Supplementary Figure 10. ^{1}H NMR spectra of PPV_{12}-*b*-$P2VP_{46}$ in CD_2Cl_2.

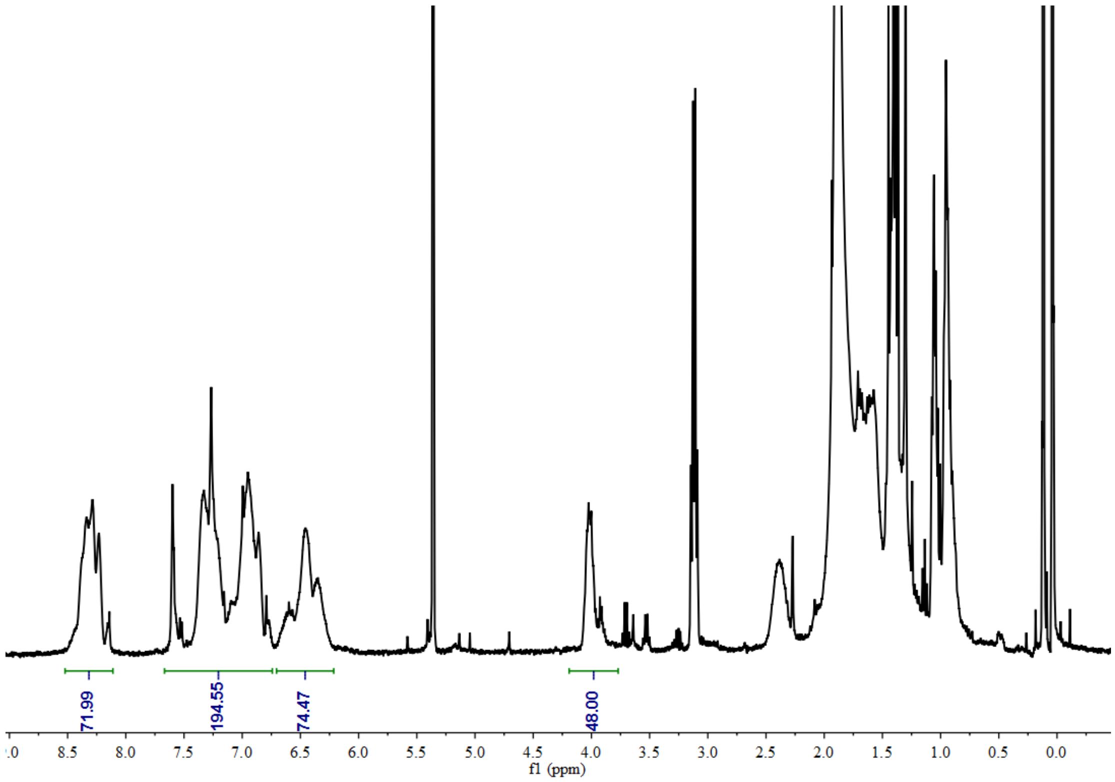

Supplementary Figure 11. ^{1}H NMR spectra of PPV$_{12}$-*b*-P2VP$_{71}$ in CD_2Cl_2.

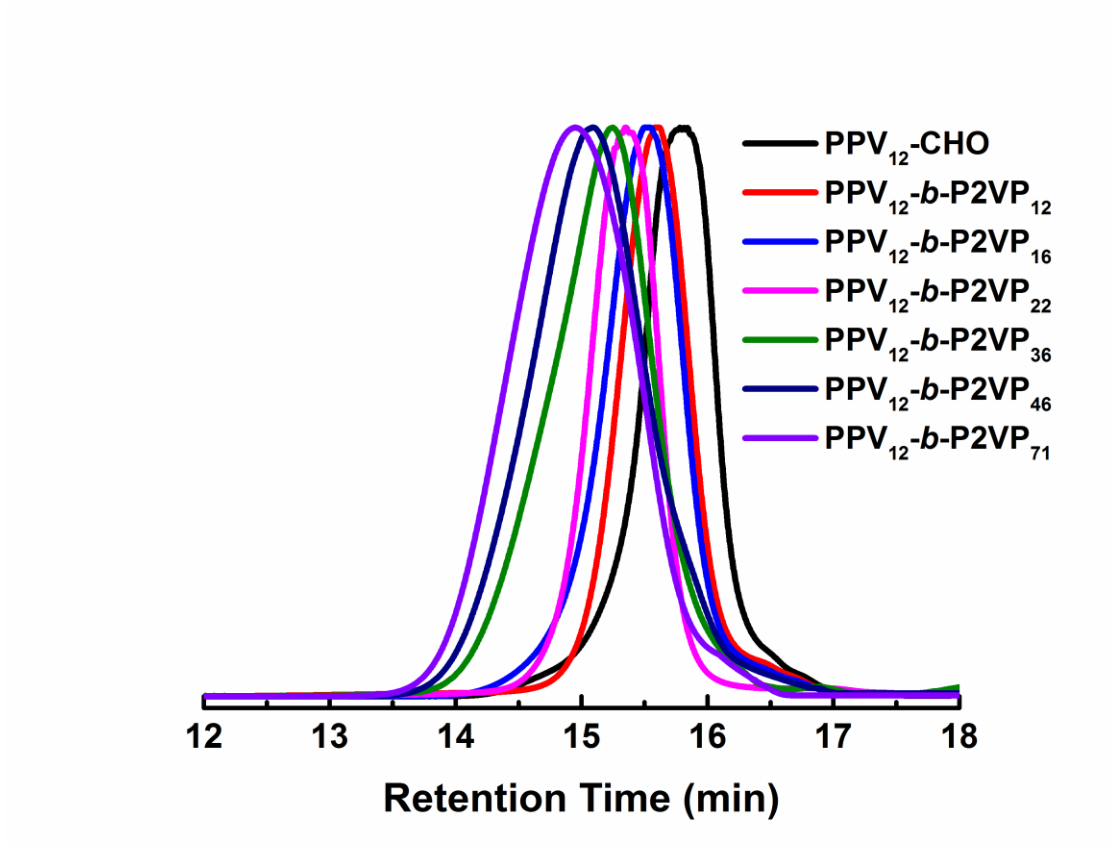

Supplementary Figure 12. GPC traces (UV-Vis) of PPV$_{12}$-CHO and BCPs PPV$_{12}$-*b*-P2VP$_{n}$.

Supplementary Table 1. Summary of the data of ^{1}H NMR and GPC of PPV_{12}-*b*-$P2VP_n$.

BCPs	block ratio of PPV/P2VP	Retention Time (min)	M_n	PDI	Integral of fields of aromatic ring in ^{1}H NMR (I_a)[a]	Number of P2VP calculated by ^{1}H NMR[b]
PPV_{12}-*b*-$P2VP_{12}$	1:1	15.58	4600	1.12	107.4	12.35
PPV_{12}-*b*-$P2VP_{16}$	1:1.3	15.52	5100	1.16	122.2	16.04
PPV_{12}-*b*-$P2VP_{22}$	1:2	15.35	6000	1.09	144.5	21.63
PPV_{12}-*b*-$P2VP_{36}$	1:3	15.25	6800	1.27	202.7	36.17
PPV_{12}-*b*-$P2VP_{46}$	1:4	15.09	8300	1.29	240.3	45.56
PPV_{12}-*b*-$P2VP_{71}$	1:6	14.95	9500	1.28	283.0	70.75

a: the integral was calculated with the integral of OCH_2 groups of PPV_{12} as standard, which was calibrated as 48; b: the number of P2VP was calculated by Supplementary Equation 1.

$$n = \frac{I_a - 48 - 10}{4} \quad (1)$$

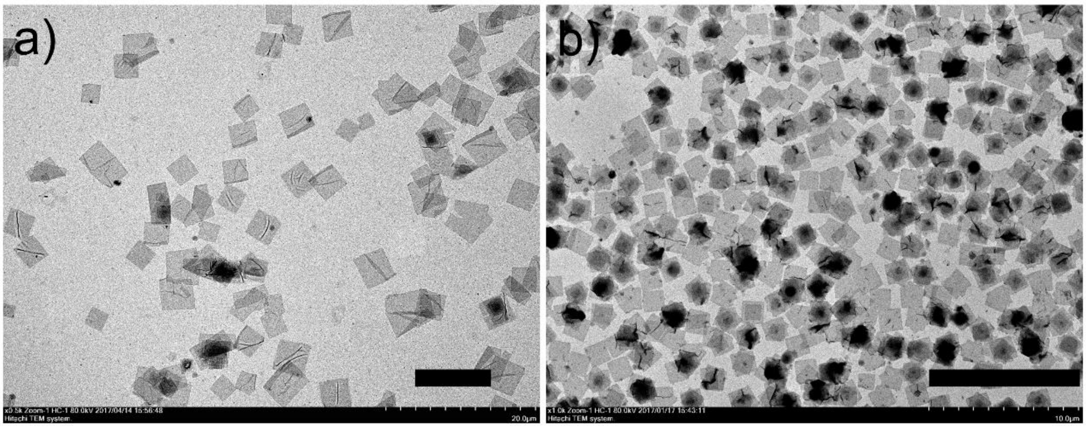

Supplementary Figure 13. The low magnification TEM micrographs of dried square micelles of PPV_{12}-*b*-$P2VP_n$ growing from isopropanol with varying block ratios. (a) PPV_{12}-*b*-$P2VP_{12}$ in 0.01 mg mL^{-1} solution; (b) PPV_{12}-*b*-$P2VP_{16}$ in 0.01 mg mL^{-1} solution. Scale bars in TEM photos are 10 μm.

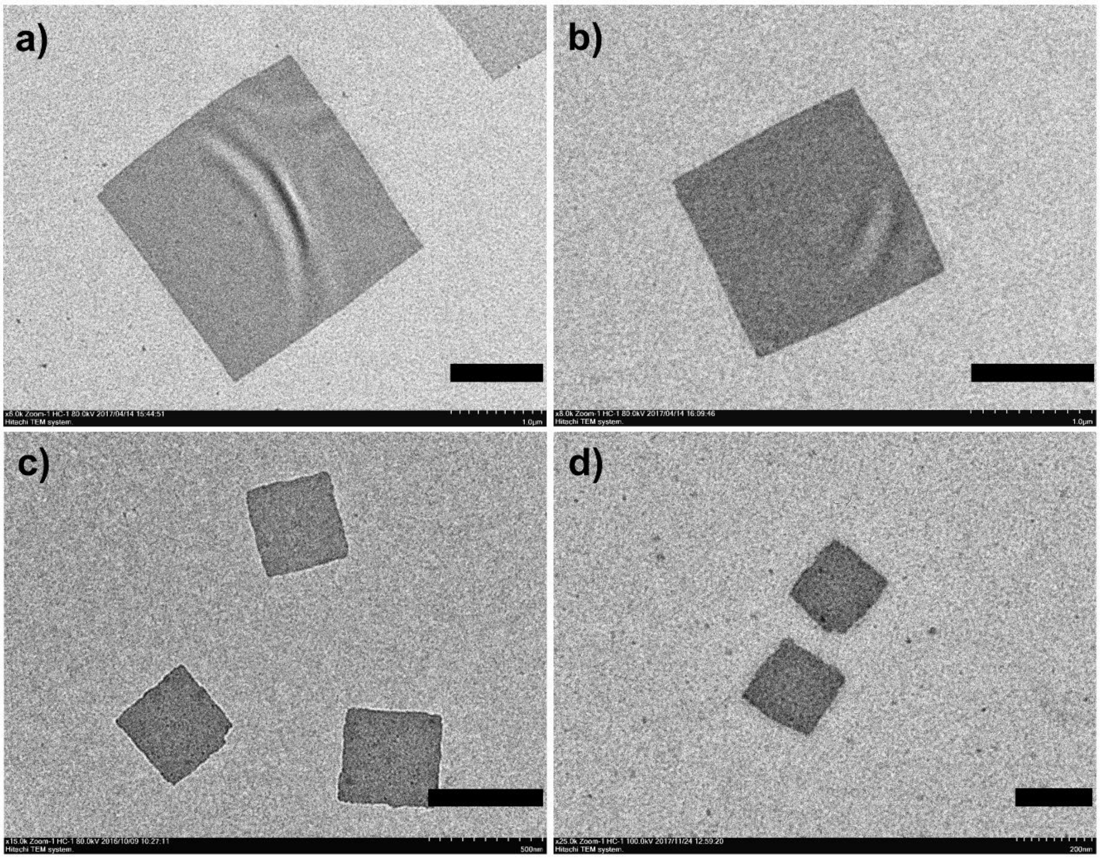

Supplementary Figure 14. The high magnification TEM micrographs of dried square micelles of PPV_{12}-*b*-$P2VP_n$ growing from isopropanol with varying block ratios. (a) PPV_{12}-*b*-$P2VP_{12}$ in 0.01 mg mL^{-1} solution, scale bar is 1 μm; (b) PPV_{12}-*b*-$P2VP_{16}$ in 0.01 mg mL^{-1} solution, scale bar is 1 μm; (c) PPV_{12}-*b*-$P2VP_{22}$ in 0.05 mg mL^{-1} solution, scale bar is 500 nm; (d) PPV_{12}-*b*-$P2VP_{36}$ in 0.05 mg mL^{-1} solution, scale bar is 200 nm.

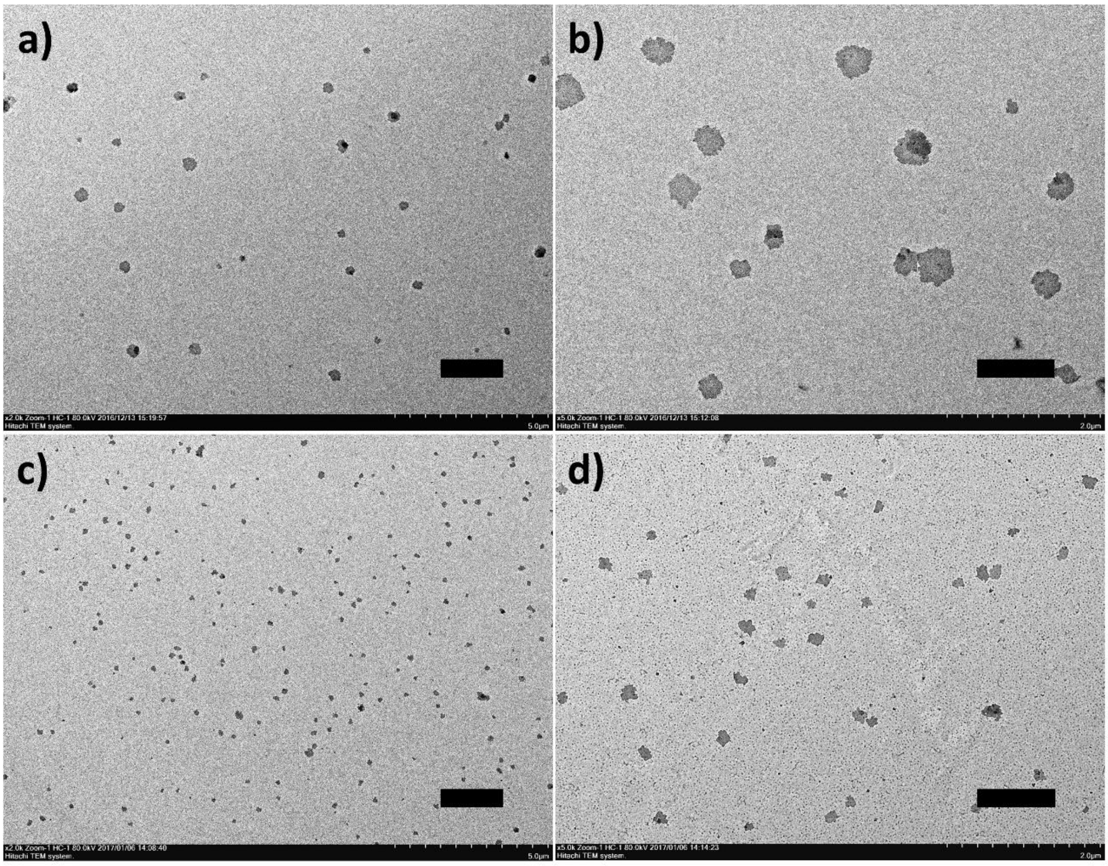

Supplementary Figure 15. The TEM micrographs of dried 2-D micelles of PPV_{12}-*b*-$P2VP_n$ growing from solution of isopropanol with varying block ratios. (a,b) PPV_{12}-*b*-$P2VP_{46}$ in 0.05 mg mL^{-1} solution; (c,d) PPV_{12}-*b*-$P2VP_{71}$ in 0.05 mg mL^{-1} solution. Scale bars in TEM photos (a, c) are 2 μm, while those in photos (b, d) are 1 μm.

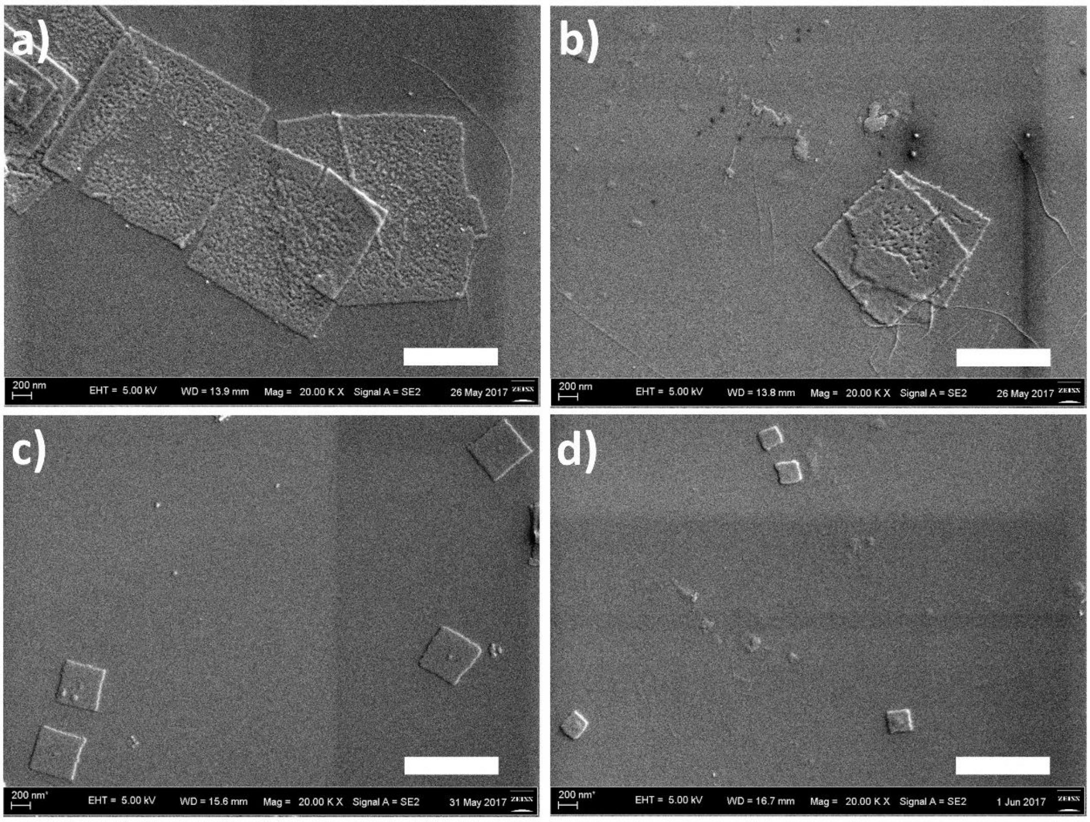

Supplementary Figure 16. The SEM micrographs of dried platelet 2-D square micelles of (a) PPV_{12}-*b*-$P2VP_{12}$ from 0.01 mg mL^{-1} isopropanol solution; (b) PPV_{12}-*b*-$P2VP_{16}$ from 0.01 mg mL^{-1} isopropanol solution; (c) PPV_{12}-*b*-$P2VP_{22}$ from 0.05 mg mL^{-1} isopropanol solution; (d) PPV_{12}-*b*-$P2VP_{36}$ from 0.05 mg mL^{-1} isopropanol solution. Scale bars in SEM photos are 1 μm.

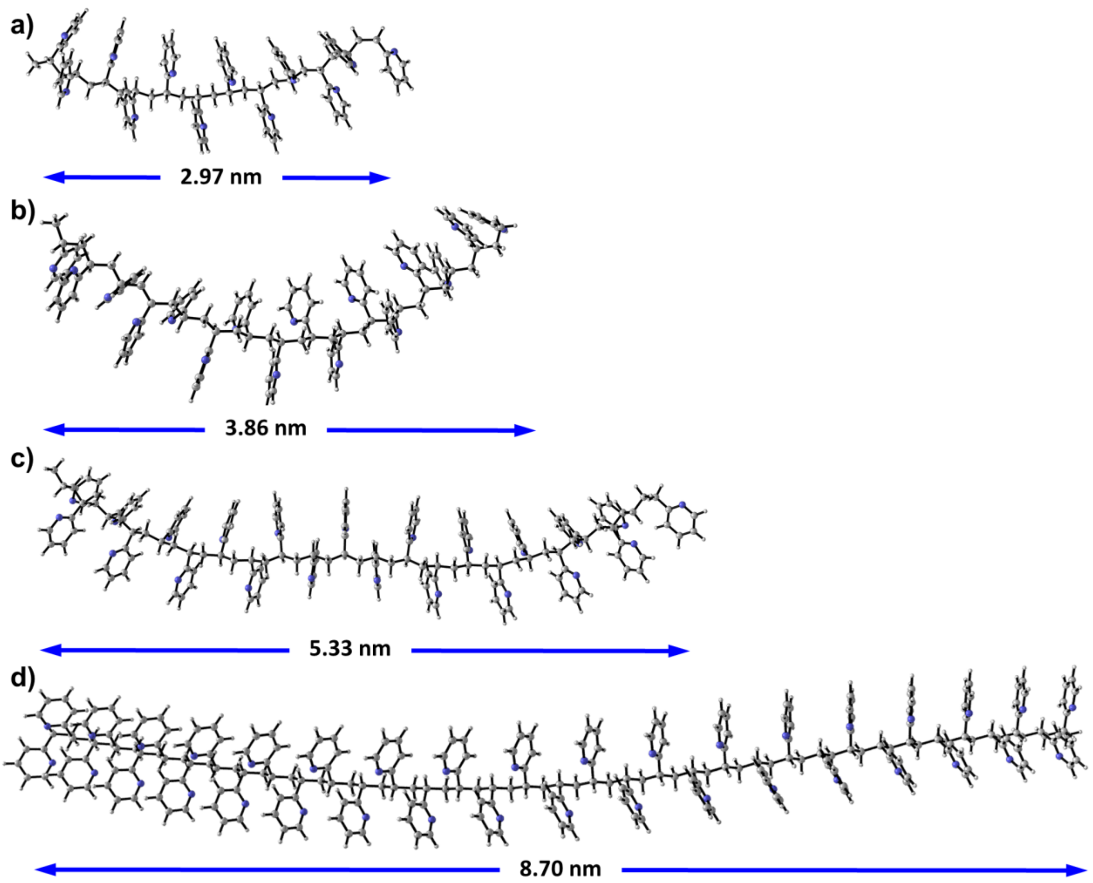

Supplementary Figure 17. The optimized structures and calculated scales of corona block (a) $P2VP_{12}$, (b) $P2VP_{16}$, (c) $P2VP_{22}$ and (d) $P2VP_{36}$, respectively, by DFT calculations. The DFT calculation was performed with the Gaussian09 suite of programs. The structure was optimized at the B3LYP level of theory with the 6-31G* basis set.

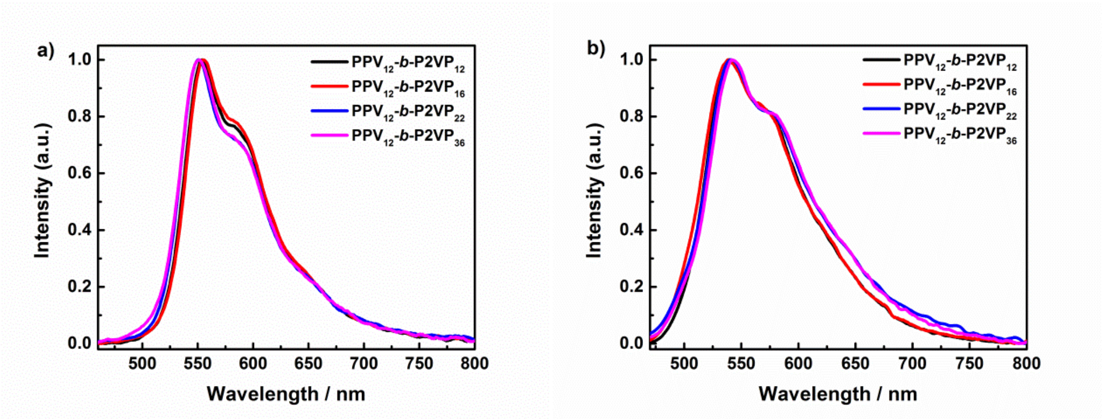

Supplementary Figure 18. The fluorescence spectra of platelet 2-D square micelles of PPV_{12}-*b*-$P2VP_n$ in 0.01 mg mL^{-1} (a) THF and (b) 2-PrOH colloidally stable solutions.

Supplementary Table 2. Summary of the data of fluorescence properties of PPV$_{12}$-*b*-P2VP$_n$ in 0.01 mg mL^{-1} THF and 2-PrOH solutions[a].

Polymers	$\lambda_{max,THF}$ (nm)	$\lambda_{max,2\text{-}PrOH}$ (nm)	$\Phi_{F,THF}$	$\Phi_{F,2\text{-}PrOH}$
PPV$_{12}$-*b*-P2VP$_{12}$	554	540	0.27	0.10
PPV$_{12}$-*b*-P2VP$_{16}$	555	539	0.27	0.09
PPV$_{12}$-*b*-P2VP$_{22}$	551	541	0.28	0.17
PPV$_{12}$-*b*-P2VP$_{36}$	551	542	0.27	0.14

[a]the PL spectra and quantum yields measured in THF and 2-PrOH solutions of the concentrations of 0.01 mg mL^{-1}, excited at 440 nm.

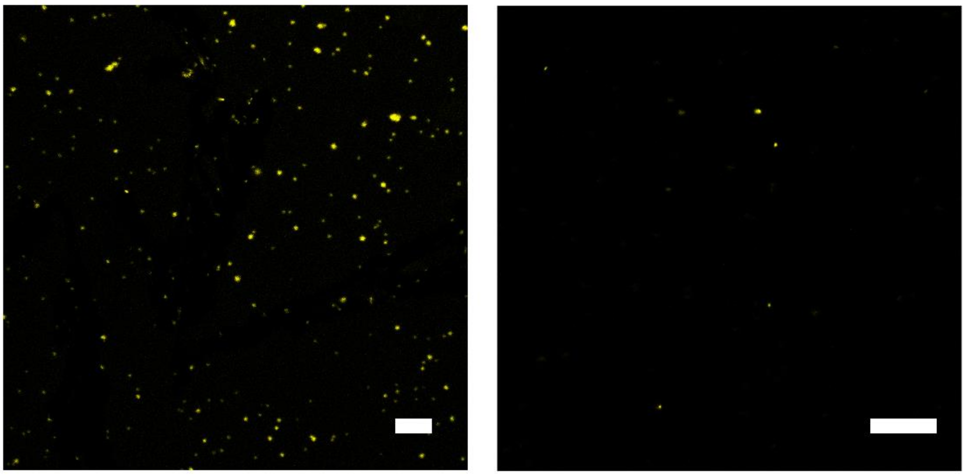

Supplementary Figure 19. LSCM images of representative sample of 2-D square micelles achieved in 0.02 mg mL^{-1} 2-PrOH solution of PPV$_{12}$-*b*-P2VP$_{22}$. Scale bars in are 5 μm.

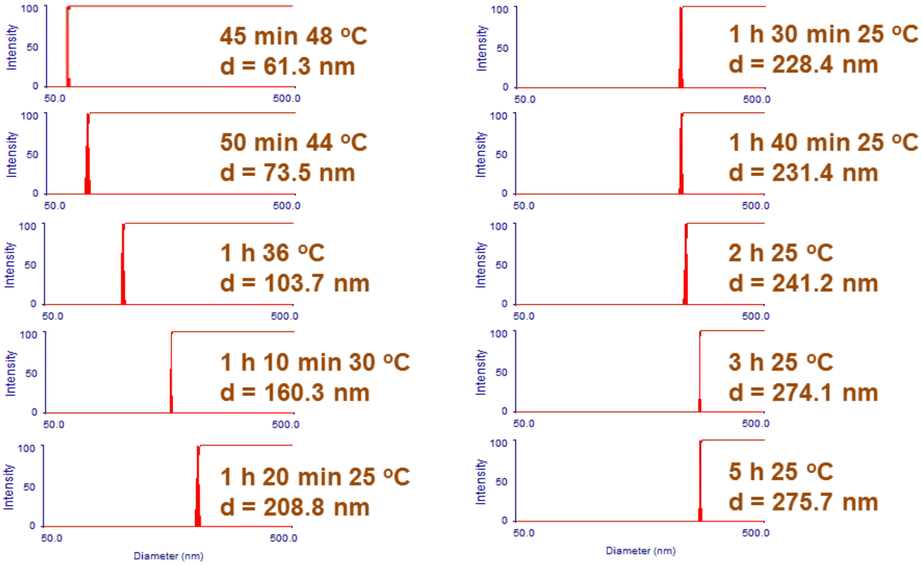

Supplementary Figure 20. The DLS results of PPV$_{12}$-*b*-P2VP$_{22}$ in 0.02 mg mL^{-1} isopropanol solutions at different temperature and aging time after being heated at 85 °C.

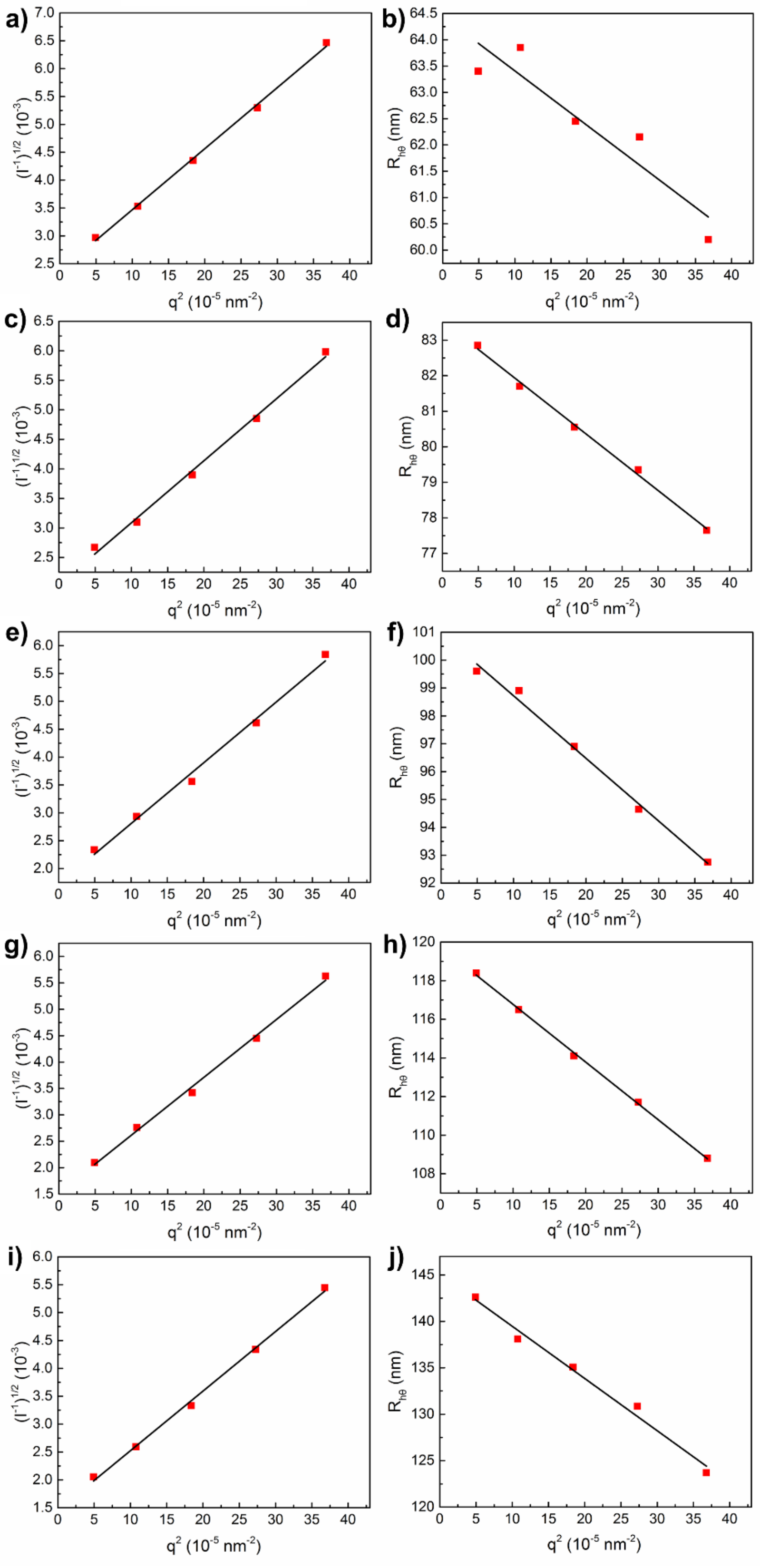

Supplementary Figure 21. The morphology factor R_g/R_h (ρ) values of PPV_{12}-*b*-$P2VP_{22}$ in 0.02 mg mg mL-1 isopropanol solutions at (a,b) 45 °C; (c,d) 40 °C; (e,f) 35 °C; (g,h) 30 °C; (i,j) 25 °C after being heated at 85 °C were calculated to be (a,b) 2.59; (c,d) 2.17; (e,f) 2.00; (g,h) 1.74; (i,j) 1.41,

respectively. The mean-square radius of gyration (R_g, images a,c,e,g,i) was calculated from SLS and determined through Berry Plot, and the hydrodynamic radius (R_h, images b,d,f,h,j) was calculated by measuring $D_{h\theta}$ of the sample solution at different angles by DLS and extrapolating the value when the angle is 0°.

Supplementary Table 3. Summary of the data of light scattering (LS) of PPV$_{12}$-*b*-P2VP$_{22}$ in 0.05 mg mL^{-1} 2-PrOH solutions[a].

Temperature (°C)	R_g[b] (nm)	R_h[c] (nm)	ρ (R_g/R_h)
45	166.8	64.4	2.59
40	181.7	83.5	2.17
35	201.8	100.9	2.00
30	209.0	119.8	1.74
25	204.0	145.1	1.41
25 after 2d	196.8	144.9	1.36

[a]the LS data measured in 0.05 mg mL^{-1} 2-PrOH solutions of PPV$_{12}$-*b*-P2VP$_{22}$ at different angles and temperature, the wavelength of incident laser was 637.0 nm. [b]R_g value was calculated from SLS at different angles (30°, 45°, 60°, 75° and 90°) and determined through Berry Plot. [c]R_h value was calculated from DLS at different angles (30°, 45°, 60°, 75° and 90°) and extrapolated when the angle is 0°.

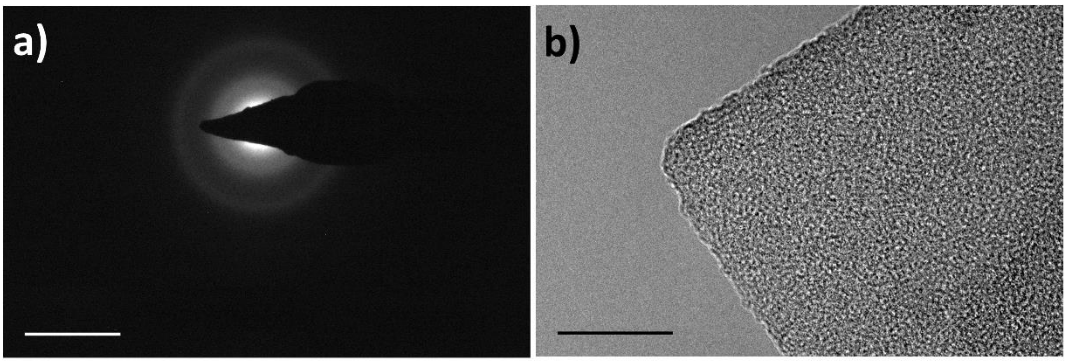

Supplementary Figure 22. (a) The selected area electron diffraction (SAED) pattern, whose scale bar is 10 nm^{-1}, and (b) high resolution TEM photograph, whose scale bar is 20 nm, of dried 2-D platelet micelles growing from 0.02 mg mL^{-1} isopropanol solutions of PPV$_{12}$-*b*-P2VP$_{22}$.

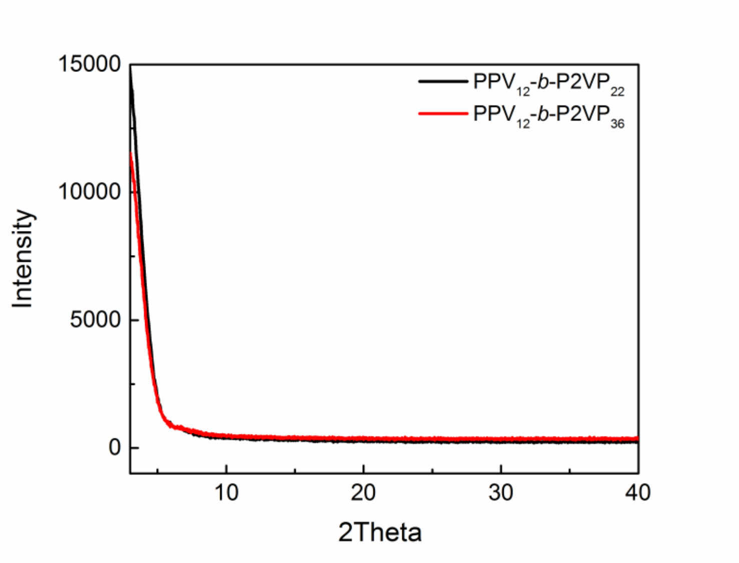

Supplementary Figure 23. WXRD profiles of platelet 2-D square micelles obtained from (a) PPV_{12}-*b*-$P2VP_{22}$ in 0.05 mg mL^{-1} 2-PrOH solution and (b) PPV_{12}-*b*-$P2VP_{36}$ in 0.05 mg mL^{-1} 2-PrOH solution.

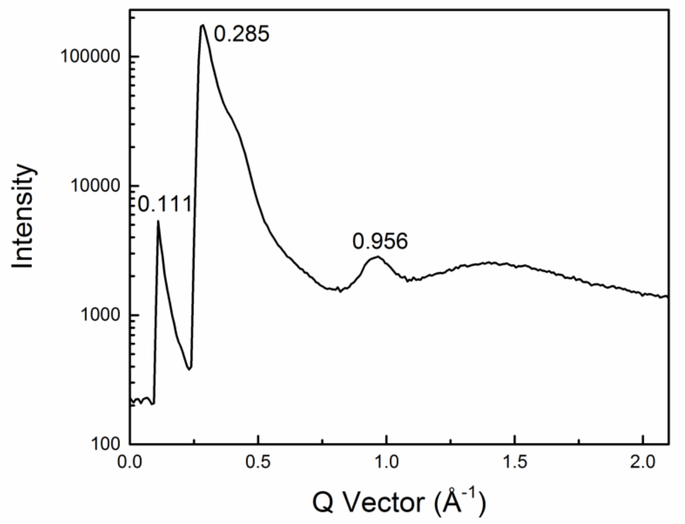

Supplementary Figure 24. GIWAXS profiles along the out-plane direction of platelet 2-D square micelles obtained from PPV_{12}-*b*-$P2VP_{22}$ in 0.05 mg mL^{-1} 2-PrOH solution.

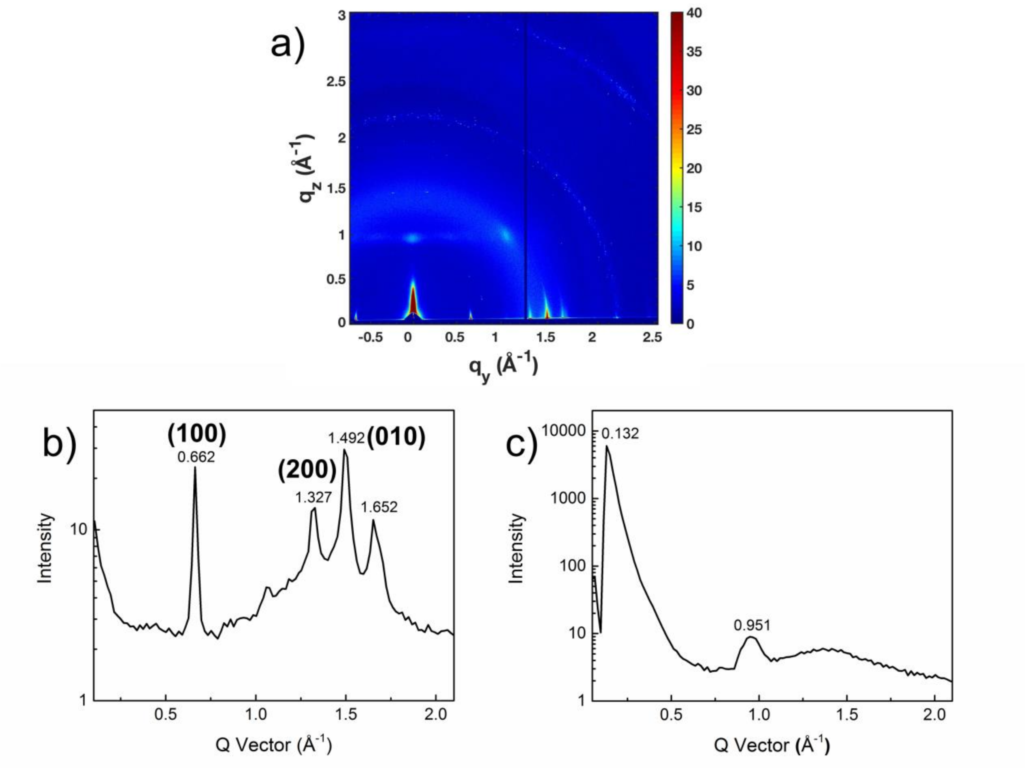

Supplementary Figure 25. (a) 2D GIWAXS patterns and GIWAXS profiles along the (b) in-plane and (c) out-plane direction of platelet 2-D square micelles obtained from PPV$_{12}$-*b*-P2VP$_{36}$ in 0.05 mg mL^{-1} 2-PrOH solution.

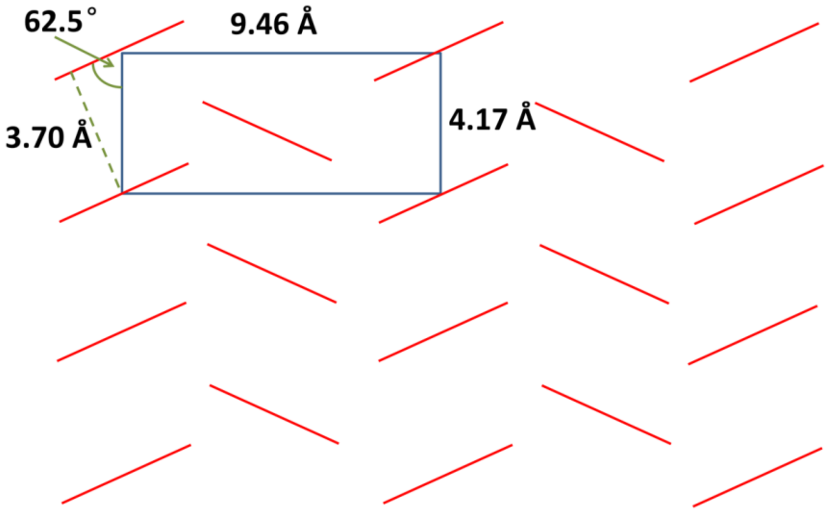

Supplementary Figure 26. The probably herringbone molecular packing in platelet 2-D square micelles calculated form GIWAXS measurement perpendicular to substrate along q_z direction. The red solid line represents the PPV$_{12}$ backbone that is vertically overlooked.

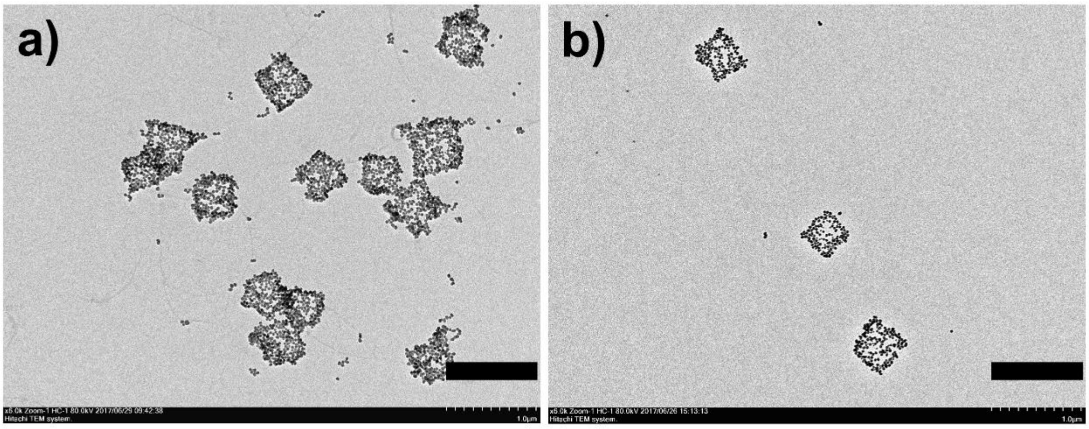

Supplementary Figure 27. (a,b) TEM images of platelet 2-D square micelles of PPV_{12}-*b*-$P2VP_{22}$, which was obtained from 0.05 mg mL^{-1} 2-PrOH solution, with silica nanoparticles selectively deposited on the P2VP coronas. Scale bars are 1 μm.
