# Supplementary material for: Uniform two-dimensional square assemblies from conjugated block copolymers driven by π–π interactions with controllable sizes
Source: Nat Commun. 2018 Feb 28;9:865. doi: 10.1038/s41467-018-03195-y (PMC5830438; doi:10.1038/s41467-018-03195-y)
Supplement: Supplementary file 2 — Description of Additional Supplementary Files [file 41467_2018_3195_MOESM2_ESM.pdf]

Description of Additional Supplementary Files

File Name: Supplementary Movie 1
Description: Consecutive LSCM images of moving fluorescent platelet 2-D square micelles in solution
